# Risk of cancer in patients with genital warts: A nationwide, population-based cohort study in Taiwan

**DOI:** 10.1371/journal.pone.0183183

**Published:** 2017-08-14

**Authors:** Ching-Yi Cho, Yu-Cheng Lo, Miao-Chiu Hung, Chou-Cheng Lai, Chun-Jen Chen, Keh-Gong Wu

**Affiliations:** 1 Department of Pediatrics, Taipei Veterans General Hospital and National Yang-Ming University, Taipei, Taiwan; 2 Division of Infectious Diseases, Department of Pediatrics, Taipei Veterans General Hospital and National Yang-Ming University, Taipei, Taiwan; Universidade Estadual de Maringa, BRAZIL

## Abstract

**Background:**

Condyloma acuminata currently affects around 1% of sexually active adults, and its incidence is increasing. The coexistence of genital warts (GW) and certain cancers and an association between human papillomavirus (HPV) and various malignancies have been reported. Therefore, we conducted this large national study to analyze the risk of malignancies among men and women with GW in Taiwan.

**Methods and findings:**

Between January 2000 and December 2013, approximately 3 million patients were reported to the National Health Insurance Research Database of Taiwan. Of these patients, 21,763 were diagnosed with GW. In the same time period, a total of 213,541 cancer cases were reported to the registry, of which 1002 were recorded among patients with GW. The age-specific incidence rates of GW and standardized incidence ratios (SIRs) of malignancies compared to the general population were calculated. Women acquired GW earlier than men, with a mean age at diagnosis of 32.63±12.78 years. The highest incidence rate for both genders peaked at 20–29 years. Of the 1002 patients with GW and malignancies, the SIR was 1.95 (95%CI 1.83–2.07). The most markedly increased risk was found for HPV-related cancers, with a SIR of 9.74 (95%CI 3.70–15.77). Significantly elevated risks were also noted for smoking-related cancers, anogenital cancers, cervix in situ, colon, rectum, lung, kidney, and prostate cancers. Most cancers developed within 10 years after the diagnosis of GW.

**Conclusions:**

Patients with GW have an increased risk of HPV-related cancers, especially anogenital malignancies in Taiwan. The elevated risk of other cancers highlights differences in exposure and risk factors among patients with GW compared to the general population. Cancer screening and HPV vaccination programs should be emphasized for at-risk patients.

## Introduction

Condyloma acuminata, also known as “genital warts”, is a common disease, affecting approximately 1% of sexually active adults worldwide [1]. Around 1.4 million people in the United States have genital warts and the incidence is increasing, with more than 500,000 new cases being reported every year. The highest incidence of genital warts has been reported in people aged 20 to 29 years [2,3].

Genital warts were first regarded to be a venereal disease, and the nature of the disease and the transmission pathway were not clarified until the 1950s and 1960s. The relationship between genital warts and a viral etiology was established in 1968 when viral particles were identified in lesions [4]. The clinical importance of the different strains of human papillomavirus (HPV) has also been reported. HPV type 6 and 11 are not carcinogenic and can cause genital warts, whereas type 16 and 18 are associated with cervical intraepithelial neoplasia and other invasive cancers [5].

Most HPV infections are benign, transient, and treatable. However, the coexistence of condyloma acuminate and malignancies of anogenital sites and head and neck regions have been reported [6–13]. It has also been hypothesized that patients with genital warts and malignancies are more likely to harbor oncogenic types of HPV [14,15].

Most researches have focused on the relationship between genital warts and cancers of anogenital and head and neck areas. Over the past 20 years, only four cohort studies have analyzed the association between genital warts and malignancies [14,16,17]. The first of these studies was conducted in Sweden in 1991, and showed no significant correlation between genital warts and cancers among women, although the number of genitourinary cancers among women with genital warts was three times higher than expected [17]. A second Swedish study published in 2006 indicated that condyloma acuminate was strongly associated with an increased risk of cancers of the vulva, vagina, penis, and anus, as well as non-anogenital malignancies [14]. A Danish study published in 1997 enrolled women only, and the results supported that genital warts significantly increased the risk of anogenital neoplasias, particularly vulvar cancer. Finally, the most recent and largest study also conducted in Denmark demonstrated a strong association between anogenital and head and neck cancers and genital warts [18].

The incidence of genital warts in Asia is also increasing [19]. Most patients tend to seek non-standard treatment or traditional therapies, and therefore many patients have a delayed diagnosis or receive inappropriate treatment. Whether patients with genital warts have an increased risk of persistent infections and malignancies has not been investigated. In Taiwan, the overall incidence of genital warts has been estimated to be around 55-65/100,000 population, with approximately 70% of the case being female with a mean age of 33 years [19–21]. This study aimed to analyze the relative risk of developing malignancies among Taiwanese men and women diagnosed with genital warts. The age distribution and duration from the diagnosis of genital warts to the development of malignancies were also documented. This is by far the largest population-based, longitudinal study in this field of research, not only in Taiwan, but also in the Asia-Pacific region. The results may play a role in promoting prevention strategies through prophylactic HPV vaccination programs and regular screening for specific cancers.

## Material and methods

### Data source

**Fig 1:**
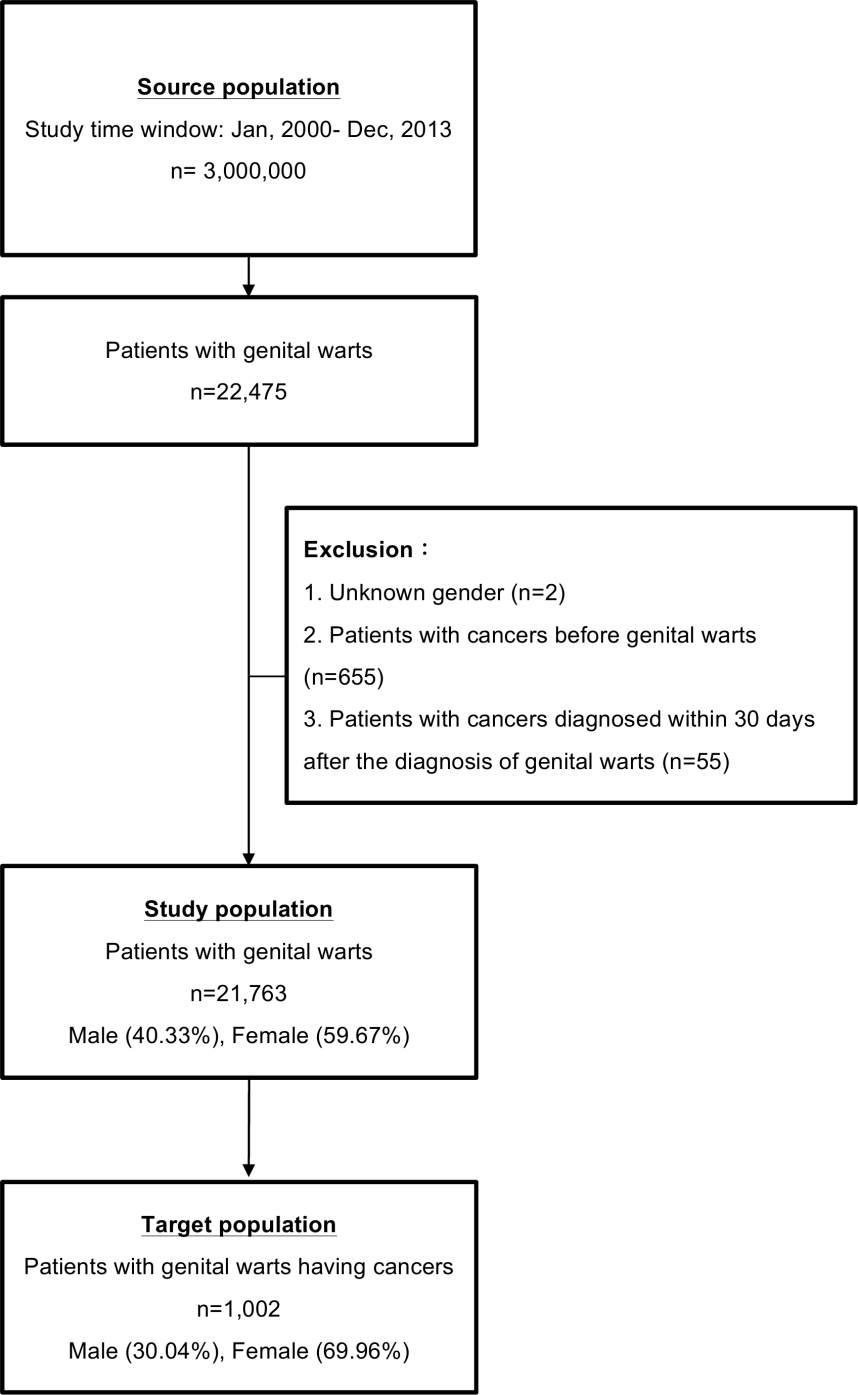
Enrollment flowchart showing the steps in this study.

Taiwan launched the National Health Insurance program in March 1, 1995. As of 2014, 99.9% of Taiwanese residents were enrolled (of a total population of around 23.5 million) [22]. The National Health Insurance Research Database (NHIRD) is comprised of registration files and original claims data including personal information, disease coding, and details of outpatient and inpatient clinic visits for all patients covered by the National Health Insurance program. To ensure the accuracy of the claims files, quarterly expert reviews on a random sample for every 50 to 100 ambulatory and inpatient claims are performed by the Bureau of National Health Insurance. Therefore, data obtained from the NHIRD are considered to be complete and accurate. The study population was derived from the Longitudinal Health Insurance Database (LHID), a subset of the NHIRD. We used data from three sets of the LHID; the LHID 2000, 2005, and 2010. Each consisted of the original claims data of one million enrollees randomly sampled from the 2000, 2005, 2010 registries from January 2000 to December 2013 (Fig 1).

### Study population

Only patients diagnosed with genital warts during the study period were enrolled. In the NHIRD, all diagnoses are coded according to International Classification of Diseases, Ninth Revision (ICD-9) codes. The outpatients diagnosed with an ICD-9 code of 078.11 (condyloma acuminatum) or 078.1 (viral warts) plus a record of a urological/gynecological clinic visit were recruited. To eliminate potential selection and surveillance bias and to ensure that the patients were diagnosed with cancers after the diagnosis of condyloma acuminata, the patients with a previous diagnosis of cancer or a cancer diagnosed within 30 days after the first diagnosis of genital warts were excluded. Cases of malignancies were also identified according to ICD-9 codes (140–239) (S1 Table).

A total of 21763 subjects were identified and enrolled as the study group (Fig 1). The comparison group was the general populations matched for age and gender with the study population.

### Target population

We identified patients with cancers among the study population (patients with genital warts) and the source population during the study period (January 2000 to December 2013). To prevent overestimation of the number of malignancies, only patients hospitalized with a catastrophic illness certificate were enrolled. We further grouped the patients with anus (ICD-9: 154.2, 154.3, 154.8), vulva (ICD-9: 184.1,184.2, 184.3, 184.4), vagina (ICD-9: 184.0), cervix (ICD-9: 180), and penis (ICD-9: 187.1, 187.2, 187.3, 187.4, 187.7, 187.8) cancer as having HPV-related cancers for the analysis using the scheme proposed by Ryerson et al. We also grouped the following as smoking-related cancers: buccal cavity and pharynx (ICD-9: 149), esophagus (ICD-9: 150), stomach (ICD-9: 151), liver (ICD-9: 155), pancreas (ICD-9: 157), larynx (ICD-9: 161), lung (ICD-9: 162), cervix uteri (ICD-9: 180), kidney (ICD-9: 189), urinary bladder (ICD-9: 188), and myeloid leukemia (ICD-9: 205) [23–25].

### Statistic analysis

The source population was followed until withdrawal from the insurance program or 31^st^ December 2013, whichever came first. Because the observation time differed between patients and the population at risk varied with time and age distribution, person-years rather than just years were used for the analysis to overcome these difficulties and for age adjustments. Age-specific incidence rates of genital warts and cancers were derived by dividing the new cases diagnosed at a specific age by the total person-years of the population of the same age. Standardized incidence ratios (SIRs), which served as the reference for the relative risk, and 95% confidence intervals (CIs) were calculated. SIRs were obtained by dividing the observed number of cases of cancers after a diagnosis of genital warts by the expected number of cases. The expected number of cases was calculated by summing the post-genital warts person-years at a specific age multiplied by the age-specific incidence of the same age of the general population with a certain cancer as follows: Expected number: $\sum_{i=0}^{n}{ASIR}_{i}\times {Person–year}_{i}$, i = at *i* years of age, n = 99, ASIR_i_ = age-specific incidence rate of the general population with a certain cancer, Person–year_i_ = total person-years of the patients with genital warts at *i* years of age. The 95% confidence level of the SIR was calculated directly from Poisson distribution when the number of observed cases was less than 100. Byar approximation was used when there were 100 cases or more.

The measured outcomes with significantly different SIRs were further stratified according to the follow-up years from the first diagnosis of genital warts to the occurrence of the malignancies (< 1 years, 1–5 years, 6–10 years, > 10 years) and stratified based on the age at the diagnosis of genital warts in 10-year intervals. P values were calculated using Pearson’sχ2 or Fisher’s exact test. A P value less than 0.05 was taken to indicate a significant difference between groups.

The study was approved by the Institutional Review Board of Taipei Veterans General Hospital (VGHIRB), Taipei, Taiwan (number 2012-06-006A).

## Results

### Risk of cancers

**Fig 2:**
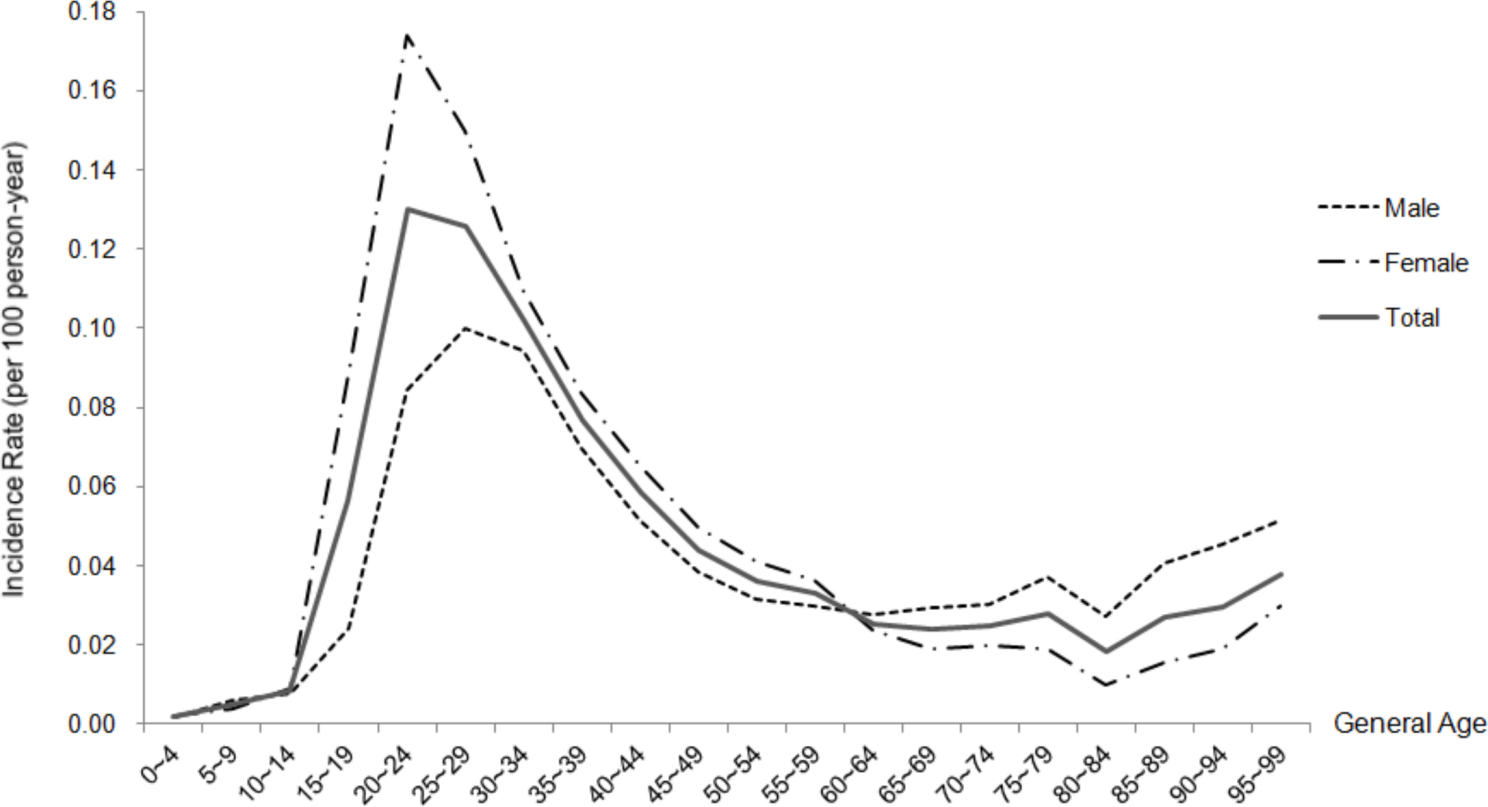
Age-specific incidence rate of genital warts. The highest incidence peaked at an age of 25–29 years for men and 20–24 years for women. Women acquired genital warts earlier than men, with a mean age at diagnosis of 34.19±13.73 years.

In total, 21763 patients (40.33% men and 59.67% women) were diagnosed with genital warts during the study period. Women acquired genital warts earlier than men by around 4 years, with a mean age at diagnosis of 32.63±12.78 years. The highest incidence of genital warts for both genders peaked at 20–29 years of age, with 0.18 cases for men and 0.32 cases for women per 100 person-years. During the follow-up period, a total of 1002 cases of cancers were identified among the patients with genital warts, with female predominance. The mean time to a diagnosis of cancer after a diagnosis of genital warts was 4.17±3.06 years (Table 1) (Fig 2, S2 Table).

**Table 1:** The characteristics of the patients with genital warts.

Characteristics	Male		Female		Total
	n	%	n	%	
Number of patients (%)	8777	40.33	12986	59.67	21763
Age at diagnosis (mean±SD)	36.51±14.71		32.63±12.78		34.19±13.73
Mean year of cancer diagnosis after genital warts^a^ (mean±SD)	3.62±2.87		4.41±3.11		4.17±3.06
Total person- year at risk^b^	47702.49	37.62	79902.64	62.38	126,795.13
Number of cancers during follow up	301	30.04	701	69.96	1002

^a^Calculated from the diagnosis of genital warts to cancer.

^b^The number of patients with genital warts was calculated from the diagnosis of genital warts to cancer, and the number of patients without genital warts was calculated from the diagnosis of genital warts until withdrawal from the insurance program or December 31, 2013.

Table 2 shows the SIRs of cancers among the patients with genital warts. Among the 1002 patients (4.60% of all genital warts patients), 301 (30.04%) were men and 701 (69.96%) were women, yielding a SIR of 1.95 (95%CI 1.83–2.07) compared to the general population. The overall excess risk was somewhat higher in men (SIR 1.87, 95%CI 1.66–2.08) than in women (SIR 1.80, 95%CI 1.66–1.93), which was mainly due to anogenital cancers (SIR 5.87, 95%CI 2.04–9.71), especially HPV-related cancers (SIR 13.57, 95%CI 3.52–23.62).

**Table 2:** Standardized incidence ratios (SIRs)^+^ of cancer among patients with genital warts.

Cancer type	Men				Women				All			
	OBS (n)	EXP (p-y)	SIR	95%CI	OBS (n)	EXP (p-y)	SIR	95%CI	OBS (n)	EXP (p-y)	SIR	95%CI
All cancers	301	160.95	1.87*	1.66–2.08	701	390.46	1.80*	1.66–1.93	1,002	514.71	1.95*	1.83–2.07
All-smoking-related cancers^a^	99	54.19	1.83*	1.47–2.19	60	31.90	1.88*	1.40–2.36	159	85.66	1.86*	1.57–2.14
All-HPV-related cancers^b^	7	0.52	13.57*	3.52–23.62	3	0.51	5.94	0.78–12.66	10	1.03	9.74*	3.70–15.77
Anogenital	9	1.53	5.87*	2.04–9.71	44	17.73	2.48*	1.75–3.21	53	18.00	2.94*	2.15–3.74
Vulva			-		2	0.17	12.05	0.65–28.75	2	0.17	12.05	0.65–28.75
Vagina					1	0.15	6.58	0.32–19.47	1	0.15	6.58	0.32–19.47
Cervix invasive					9	7.76	1.16	0.40–1.92	9	7.76	1.16	0.40–1.92
Cervix In situ					33	9.87	3.34*	2.20–4.48	33	9.87	3.34*	2.20–4.48
Anal	3	0.25	11.95	0.57–25.48	0	0.20			3	0.45	6.70	0.88–14.27
Penis	4	0.27	14.87	0.30–29.44					4	0.27	14.87	0.30–29.44
Testis	2	1.04	1.93	0.74–4.60			-		2	1.04	1.93	0.74–4.60
Head and Neck	14	14.86	0.94	0.45–1.44	1	2.38	0.42	0.40–1.25	15	18.78	0.80	0.39–1.20
Lip	1	0.75	1.34	0.28–3.96	0	0.05			1	0.89	1.13	0.08–3.34
Gum	2	1.62	1.23	0.48–2.94	0	0.12			2	1.93	1.04	0.40–2.48
Tongue	2	4.57	0.44	0.17–1.04	1	0.74	1.35	0.30–4.00	3	5.89	0.51	0.07–1.08
Mouth	8	6.73	1.19	0.36–2.01	0	0.43			8	8.00	1.00	0.31–1.69
Tonsils	1	1.81	0.55	0.53–1.64	0	0.27			1	2.29	0.44	0.42–1.29
Nasal cavities, middle ear, sinuses	1	0.40	2.48	0.38–7.34	0	0.27			1	0.70	1.44	0.38–4.25
Salivary glands	1	0.43	2.31	0.22–6.85	0	0.57			1	0.96	1.04	0.00–3.08
Pharynx	0	0.19			0	0.02			0	0.22		
Larynx	1	1.50	0.67	0.64–1.98	0	0.10			1	1.60	0.63	0.60–1.85
Esophagus	6	4.06	1.48	0.30–2.66	0	0.28			6	4.63	1.29	0.26–2.33
Stomach	8	5.66	1.41	0.43–2.39	4	3.51	1.14	0.02–2.26	12	8.86	1.35	0.59–2.12
Intestine	0	0.58			1	0.39	2.54	0.44–7.51	1	0.99	1.01	0.97–3.00
Colon	13	9.23	1.41	0.64–2.17	15	7.28	2.06*	1.02–3.10	28	16.30	1.72*	1.08–2.35
Rectum	13	6.79	1.92	0.87–2.96	6	4.61	1.30	0.26–2.34	19	11.33	1.68	0.92–2.43
Liver	29	19.28	1.50	0.96–2.05	19	6.56	2.90*	1.59–4.20	48	26.93	1.78*	1.28–2.29
GB and biliary tract	0	1.16			3	0.77	3.90	0.51–8.30	3	1.93	1.55	0.20–3.31
Pancreas	4	2.12	1.89	0.04–3.74	4	1.30	3.07	0.06–6.08	8	3.42	2.34	0.72–3.96
Lung	37	14.20	2.61*	1.77–3.45	14	7.72	1.81	0.86–2.76	51	21.00	2.43*	1.76–3.10
Melanoma	0	0.28			0	0.33			0	0.58		
Nonmelanoma skin cancer	4	1.69	2.36	0.05–4.68	1	1.24	0.81	0.78–2.39	5	2.95	1.70	0.21–3.18
Breast woman			-		48	35.28	1.36	0.98–1.75	48	35.28	1.36	0.98–1.75
Breast man	0	0.10							0	0.10		
Corpus uteri	0	0.00			9	7.76	1.16	0.40–1.92	9	7.21	1.25	0.43–2.06
Ovary, fallopian tube, broad ligament	0	0.00			9	5.58	1.61	0.56–2.67	9	4.86	1.85	0.64–3.06
Prostate	18	8.95	2.01*	1.08–2.94			-		18	8.95	2.01*	1.08–2.94
Kidney	9	3.56	2.53	0.88–4.18	6	2.59	2.32	0.46–4.18	15	6.23	2.41*	1.19–3.63
Urinary bladder	8	4.86	1.65	0.51–2.79	1	1.70	0.59	0.56–1.74	9	6.36	1.42	0.49–2.34
CNS (meninges, brain, spinal cord)	1	1.71	0.59	0.56–1.74	4	1.74	2.30	0.05–4.56	5	3.55	1.41	0.17–2.64
Hodgkin lymphoma	0	0.35	-		0	0.65			0	1.01		
Non- Hodgkin lymphoma	9	3.41	2.64	0.91–4.36	4	3.19	1.25	0.03–2.48	13	6.64	1.96	0.89–3.02
Multiple myeloma	1	0.67	1.49	0.43–4.42	0	0.44			1	1.10	0.91	0.87–2.69
Leukemia	2	2.45	0.82	0.31–1.95	5	2.32	2.15	0.27–4.04	7	4.92	1.42	0.37–2.48

SIR, standardized incidence ratios; OBS, observed; EXP, expected; CI, confidence interval; p-y, person-years; HPV, human papillomavirus; GB, gall bladder; CNS, central nervous system.

^+^SIR = $\sum_{i=0}^{n}{ASIR}_{i}\times {Person–year}_{i}$.

i = at *i* years of age, n = 99, ASIR_i_ = age-specific incidence rate of the general population with a certain cancer, Person–year_i_ = total person-years of a genital warts patient at *i* years of age.

^a^ Buccal cavity and pharynx (ICD-9 149), esophagus (150), stomach (151), liver (155), pancreas (157), larynx (161), lung (162), cervix uteri (180), kidney (189), urinary bladder (188), myeloid leukemia (205).

^b^Anus (ICD-9 154.2, 154.3,154.8), vulva (184.1,184.2, 184.3, 184.4), vagina (184.0), cervix (180), penis (187.1, 187.2, 187.3, 187.4, 187.7, 187.8)

* Significant SIR compared to the general population.

The most marked increased risk was noted for HPV-related cancers in men, with a SIR of 13.57 (95%CI 3.52–23.62), specifically anogenital cancers, (SIR 5.87, 95%CI 2.04–9.71) which was mainly due to anal and penis malignancies. In women, a significantly higher risk of anogenital cancers was noted in cervix in situ (SIR 3.34, 95%CI 2.20–4.48).

Few head and neck cancers were identified, with 14 in men and 1 in a woman, none of whom had a markedly elevated SIR. When analyzing smoking-related cancers, including cancers of the buccal cavity, pharynx, larynx, esophagus, lung, stomach, liver, pancreas, cervix uteri, kidney, urinary bladder and myeloid leukemia, both genders had significantly increased risks compared to the general population, with SIRs of 1.83 in men (95%CI 1.47–2.19) and 1.88 in women (95%CI 1.40–2.36). More detailed analysis revealed a markedly increased SIR for lung cancer in men (SIR 2.0, 95%CI 1.4–2.8) and kidney cancer in women (SIR 2.41, 95%CI 1.19–3.63), which made an important contribution to the risk estimates of smoking-related cancers.

Moreover, moderately increased risks were also noted for liver cancer (SIR 1.78, 95%CI 1.28–2.29), colon cancer (SIR 1.72, 95%CI 1.08–2.35), and prostate cancer (SIR 2.40, 95%CI 1.29–3.50) after a diagnosis of genital warts. The risk of almost all of the others cancers did not differ from that expected in the general population.

### Cancers stratified by length of follow-up and age at a diagnosis of genital warts

Table 3 illustrates the cases of cancers with significantly increased SIRs stratified by the duration of follow-up and the age at a diagnosis of genital warts. Of the patients with smoking-related cancers, 77.18% had malignancies within 5 years of the diagnosis of genital warts. The age at a diagnosis of genital warts was distributed across all age groups. All of the patients with HPV-related cancers developed malignancies within 10 years, and approximately 70% of these patients were diagnosed with genital warts at 40–49 years of age. Similarly, all of the patients with anogenital cancers were diagnosed within 10 years, and 54.72% of these patients had a diagnosis of genital warts before 49 years of age.

**Table 3:** Selected sites of cancers according to follow-up time/ age at diagnosis of genital warts. ^+^

Time^§^/Age^#^ by cancer site (Case number)	All-smoking -related cancers^a^	All-HPV -related cancers^b^	Anogenital	Cervix In situ	
Total case number	159	10	53	33	
Follow-up time^§^ (years)					
<1	29 (18.24%)	2 (20%)	18 (33.96%)	11 (33.33%)	
1–5	93 (58.49%)	6 (60%)	22 (41.51%)	12 (36.36%)	
6–10	31 (19.5%)	2 (20%)	13 (24.53%)	10 (30.3%)	
>10	6 (3.77%)	0 (0%)	0 (0%)	0 (0%)	
P value^∞^	<0.001*	0.246	<0.001*	<0.001*	
Age^#^ (years old)					
10~19	1 (0.63%)	1 (10%)	2 (3.77%)	1 (3.03%)	
20~29	6 (3.77%)	0 (0%)	13 (24.53%)	10 (30.3%)	
30~39	25 (15.72%)	0 (0%)	14 (26.42%)	11 (33.33%)	
40~49	32 (20.13%)	7 (70%)	15 (28.3%)	8 (24.24%)	
50~59	25 (15.72%)	0 (0%)	4 (7.55%)	3 (9.09%)	
60~69	24 (15.09%)	0 (0%)	3 (5.66%)	0 (0%)	
70~79	29 (18.24%)	2 (20%)	2 (3.77%)	0 (0%)	
80~89	15 (9.43%)	0 (0%)	0 (0%)	0 (0%)	
90~99	2 (1.26%)	0 (0%)	0 (0%)	0 (0%)	
P value^∞^	<0.001*	<0.001*	0.090	0.585	
Time^§^/Age^#^ by cancer site (Case number)	Colon	Liver	Lung	Prostate	Kidney
Total case number	28	48	51	18	15
Follow-up time^§^/ (years)					
<1	6 (21.43%)	8 (16.67%)	10 (19.61%)	4 (22.22%)	1 (6.67%)
1–5	13 (46.43%)	33 (68.75%)	28 (54.9%)	9 (50%)	8 (53.33%)
6–10	8 (28.57%)	4 (8.33%)	11 (21.57%)	5 (27.78%)	6 (40%)
>10	1 (3.57%)	3 (6.25%)	2 (3.92%)	0 (0%)	0 (0%)
P value^∞^	0.056	<0.001*	0.002*	0.085	0.558
Age^#^ (years old)					
10~19	0 (0%)	0 (0%)	0 (0%)	0 (0%)	1 (6.67%)
20~29	2 (7.14%)	2 (4.17%)	0 (0%)	0 (0%)	0 (0%)
30~39	6 (21.43%)	5 (10.42%)	8 (15.69%)	0 (0%)	0 (0%)
40~49	3 (10.71%)	10 (20.83%)	10 (19.61%)	2 (11.11%)	6 (40%)
50~59	4 (14.29%)	12 (25%)	5 (9.8%)	5 (27.78%)	2 (13.33%)
60~69	6 (21.43%)	11 (22.92%)	5 (9.8%)	3 (16.67%)	2 (13.33%)
70~79	6 (21.43%)	5 (10.42%)	13 (25.49%)	5 (27.78%)	3 (20%)
80~89	1 (3.57%)	3 (6.25%)	9 (17.65%)	3 (16.67%)	1 (6.67%)
90~99	0 (0%)	0 (0%)	1 (1.96%)	0 (0%)	0 (0%)
P value^∞^	<0.001*	<0.001*	<0.001*	<0.001*	<0.001*

^+^None of the patients diagnosed with genital warts at 0–9 years of age suffered from cancers thereafter, thus the interval 0–9 was not included in the table.

^§^Follow-up time from the diagnosis of genital warts to cancers.

^#^Age at a diagnosis of genital warts.

^∞^P value (Pearson’s χ^2^ or Fisher’s exact test) <0.05.

*Statistically significant.

^a^Buccal cavity and pharynx (ICD-9 149), esophagus (150), stomach (151), liver (155), pancreas (157), larynx (161), lung (162), cervix uteri (180), kidney (189), urinary bladder (188), myeloid leukemia (205).

^b^Anus (ICD-9 154.2, 154.3,154.8), vulva (184.1,184.2, 184.3, 184.4), vagina (184.0),cervix (180), penis (187.1, 187.2, 187.3, 187.4, 187.7, 187.8).

Colon, liver, lung, prostate, and kidney cancers had a similar trend in that the malignancies often developed within 5 years. The patients with colon, liver, and lung cancers tended to have a biphasic distribution of the age at a diagnosis of genital warts, while those with prostate and kidney cancers were diagnosed with genital warts at an older age.

## Discussion

In this NHIRD-based study, we investigated a large number of patients with genital warts. The study subjects were well-stratified and the number of observed and expected cancers were reliable owing to the accurate registration in the NHIRD.

The age distribution of the patients with genital warts in the present study showed a peak incidence at 20–29 years of age, which is consistent with the results of previous reports (Hsueh et al reported that more than 65% of patients were aged 20–39 years in Taiwan in 2002 and 2003) [19].

We found a significantly increased risk of anogenital cancers (especially HPV-related cancers) in the patients with genital warts. We also observed a moderately increased risk of smoking-related cancers, lung, liver, kidney, and prostate cancers. Men with genital warts had a slightly higher risk of developing cancer at all sites compared to women.

The specific mechanism by which patients with genital warts have an elevated risk of cancer is not well understood. The first epidemiological report that indicated an association between condyloma and cancer was published 1953. After advances in DNA hybridization techniques, HPV type 16 and 18 were found in up to 90% of patients with cervical carcinoma. In addition, the high-risk types of HPV have often been detected in patients with genital warts [24,26]. The oncogenic mechanisms for the virus entering a lesion, altering the epithelium characteristics, and suppressing local immunity, thereby resulting in an increased likelihood of harboring oncogenic HPV types have been established. Some reports have also reported that infection with non-oncogenic HPV types 6 and 11, which cause condyloma acuminata, may facilitate coinfection with the oncogenic types of HPVs and the subsequent pathologic changes [14]. In the current study, most of the cases of cancers with significantly increased SIRs developed near the sites where genital warts occur (anus, penis, vulva, cervix). Thus the elevated risk of anogenital cancers (especially HPV-related ones) may be related to the oncogenic nature of HPV. However, it could also be explained by infection with both oncogenic and non-oncogenic types of HPV as they share similar exposure conditions, route of transmission, and risk factors in patients with genital warts compared with the general population.

Other factors including smoking, alcohol consumption, and behavior (e.g. male homosexuality, higher sexual activity or multiple partners) are also known to be involved in oncogenic processes and may be confounding factors. Previous studies have reported that individuals with genital warts may have higher rates of alcohol consumption, smoking, and other deleterious lifestyle habits [9]. Smoking has been shown to weaken the immune response against viral infections, and more smokers have been shown to be HPV-positive than non-smokers [27,28]. In the current study, the patients with genital warts had an obviously increased risk of developing smoking-related cancers and anogenital malignancies. However, the NHIRD does not contain data on smoking so we could not perform further analysis. Further studies eliminating these confounding factors are warranted for a more precise and comprehensive evaluation.

Although no increased risk of invasive cervical cancer was observed in the present study, cervical cancer in situ has been associated with genital warts. This could be due to the relatively short follow-up time, insufficient number of cases, or a lower risk than previously thought. In addition, since annual Pap smears have been heavily promoted for women above 35 years of age in Taiwan since 1995, many potentially invasive cancers may have been diagnosed at the in situ stage and treated early. It is also possible that because the patients with genital warts were more closely monitored, precancerous lesions or cancer in situ were detected early resulting in a lower incidence of invasive cancer. Nordenvall et al. also reported a two-fold higher risk of cervical carcinoma in situ in a Swedish cohort after the introduction of population-based Pap screening programs [14]. Hence, the intensity of medical surveillance, more liberal diagnostic criteria, and increased detection and treatment in women with genital warts may explain the lack of association with invasive cancer in this study. In Taiwan, trial-based screening programs were initiated for groups at high risk of colorectal, breast, and oral cancers as well as Pap smear tests in 1985, and outreach services up to the national level have been available since 1999. The early detection of precancerous pathological changes may partially explain the less significant association between warts and these malignancies in the present study.

HPV was first considered to be involved in oral and laryngeal squamous cell carcinogenesis in 1980 due to similarities in the morphological features between genital and oral-HPV-associated lesions [29,30]. Many subsequent studies have concluded that HPV infection is associated with the development of head and neck cancers among subjects with or without established risk factors such as tobacco and alcohol use [13,30–32]. D’Souza et al. reported that a higher incidence of HPV-associated head and neck squamous cell carcinoma was related to a higher frequency of sexual risk-taking behavior (more sexual partners and/or more oral sex) [13]. Nevertheless, Taiwan has one of the highest incidence rates of head and neck cancer in the world (annual incidence 22 per 100,000), yet there was no significantly increased risk of developing head and neck cancers in the patients with genital warts. Studies from Western countries have reported an increasing trend of HPV-related head and neck cancers despite a decrease in the overall incidence [33–35]. In contrast, the overall incidence of head and neck cancers in Taiwan continued to rise from 1995–2009, with a trend of a more rapid rise in HPV-related head and neck cancers [36]. Alcohol, tobacco, and betel quid chewing account for a large proportion of cases. The relatively high prevalence has also been correlated with genetic, virological, and molecular factors. The large patient population and lower frequency of certain sexual behaviors may have contributed to our study results of no significantly elevated risk of developing head and neck cancers after a diagnosis of genital warts.

We also found a higher SIR for nonmelanoma skin cancer. Few studies have examined the association between HPV and nonmelanoma skin cancer. The cutaneous type of HPV may contribute to oncogenesis together with genotoxic ultraviolet exposure, immunocompromised status, and tobacco use [37–39]. However, the potential association with HPV is controversial, and further research on this issue is needed. The same may be true for lung cancer. Our results showed a two-fold increased risk of lung cancer compared to the general population in men. Although cigarette smoking is considered to be the most important factor, the presence of circulating HPV DNA in the blood has been suggested to be an oncogenic risk marker for lung cancer [40]. With regards to hepatic cancer, there are currently no related studies describing an association with genital warts or HPV. The relatively high prevalence of chronic hepatitis B infection in Taiwan may also have confounded the results [41]. Wait et al. reported that there are currently around 3 million carriers of HBV in Taiwan, and that liver cancer is the leading cause of cancer deaths in Taiwanese men and the second leading cause in women. Therefore, further studies are needed to investigate the relationship between genital warts and hepatic cancer. Similar findings have been reported for colorectal, kidney and prostate cancers and the relationships between these malignancies with viral infections, especially HPV infection, are still controversial and require further studies.

Individuals with HPV infection mainly developed benign hyperplasia with low malignant potential, however a subgroup of high-risk HPVs with precancerous potential may trigger malignant changes in local tissues. A small fraction of these individuals patients develop cancers, usually many years after the initial infection. In the current study, the development of most malignancies (60–80%) occurred within 5 years after the diagnosis of genital warts. In particular, more than one third of the patients with anogenital cancers, especially cervix in situ, were diagnosed with malignancies within 1 year after the diagnosis of genital warts. The misdiagnosis of the early stage of cancers as genital warts (diagnostic bias) or the routine screening for cervical and oropharyngeal cancers in women and high-risk groups in Taiwan may explain these findings (surveillance bias). The relatively short observation period in the present study may also have played a role in the results.

In stratification by age at the diagnosis of genital warts, the patients with HPV-related cancers, anogenital cancer, and cervix in situ, tended to be diagnosed with genital warts at an earlier age (< 50 years). However the risk of the other cancers remained high in the patients with an older age at the diagnosis of genital warts, possibly because of relatively weak immunity in the elderly.

There are several limitations to this study. First, the incidence of genital warts may have been underestimated. Many cases were likely to have been underreported, particularly those diagnosed outside hospitals by clinicians not enrolled with the National Health Insurance program. Second, the majority of the enrolled subjects did not reach the age at which the incidence of cancers peaked due to the relatively short follow-up period. In addition, due to the nature of the NHIRD, survival bias should be considered. Further, the relative rarity of certain cancers may have led to the estimates being inaccurate. Finally, the study did not include data on sexual behavior, smoking, betel quit chewing, socioeconomic status, or other lifestyle habits, all of which may play important roles as confounding factors.

## Conclusions

This study demonstrated a significantly increased risk of both anogenital cancers and cancers of other sites among patients with genital warts. However, the elevated risks of smoking-related cancers, liver, lung, prostate, and kidney cancers may indicate differences in exposure and risk factors in patients with genital warts compared to the general population. Further studies taking confounders into consideration and direct detection of HPV oncogenic pathways among various cancers are needed. Since there is usually a latent period between the initial infection and the development of malignancies, Pap smears and other screening programs should be emphasized, especially in high-risk groups. Prophylactic vaccinations against oncogenic HPVs and to further block persistent infections should also be promoted. Several studies have demonstrated that vaccines can successfully elicit high levels of neutralized antibodies and reduced rates of persistent HPV infections, thereby decreasing the incidence of both cervical carcinoma and also other anogenital and HPV-related head and neck cancers [13,42]. In Taiwan, HPV vaccines received regulatory approval in October 2006 as a self-paid option for females aged 9–26 years, and substantial population benefits with a promising and favorable cost-effectiveness ratio have been shown [43]. Therefore, we suggest that governments should offer free HPV vaccines to the general population and improve the compliance rate, particularly in those at high-risk. Further studies are warranted to investigate whether HPV vaccines can protect against genital warts and cervical cancer and also against other malignancies.

## Supporting information

S1 Table ICD-9 codes for all cancers. (DOCX)

S2 Table Age-specific incidence rate of genital warts. (DOCX)
