# Supplementary material for: Risk of cancer in patients with genital warts: A nationwide, population-based cohort study in Taiwan
Source: PLoS One. 2017 Aug 14;12(8):e0183183. doi: 10.1371/journal.pone.0183183 (PMC5555692; doi:10.1371/journal.pone.0183183)
Supplement: S1 Table — (DOCX) [file pone.0183183.s001.docx]

S1 Table. ICD-9 codes for all cancers

| ICD-9 code | Topography |
| --- | --- |
| 140 | *Malignant neoplasm of lip |
| 1400 | Malignant neoplasm of upper lip, vermilion border |
| 1401 | Malignant neoplasm of lower lip, vermilion border |
| 1403 | Malignant neoplasm of upper lip, inner aspect |
| 1404 | Malignant neoplasm of lower lip, inner aspect |
| 1405 | Malignant neoplasm of lip, unspecified, inner aspect |
| 1406 | Malignant neoplasm of commissure of lip |
| 1408 | Malignant neoplasm of other sites of lip |
| 1409 | Malignant neoplasm of lip, unspecified, vermilion border |
| 141 | *Malignant neoplasm of tongue |
| 1410 | Malignant neoplasm of base of tongue |
| 1411 | Malignant neoplasm of dorsal surface of tongue |
| 1412 | Malignant neoplasm of tip and lateral border of tongue |
| 1413 | Malignant neoplasm of ventral surface of tongue |
| 1414 | Malignant neoplasm of anterior two-thirds of tongue, part unspecified |
| 1415 | Malignant neoplasm of tongue of junctional zone |
| 1416 | Malignant neoplasm of lingual tonsil |
| 1418 | Malignant neoplasm of other sites of tongue |
| 1419 | Malignant neoplasm of tongue, unspecified |
| 142 | *Malignant neoplasm of major salivary glands |
| 1420 | Malignant neoplasm of parotid gland |
| 1421 | Malignant neoplasm of submandibular gland |
| 1422 | Malignant neoplasm of sublingual gland |
| 1428 | Malignant neoplasm of other major salivary glands |
| 1429 | Malignant neoplasm of salivary gland, unspecified |
| 143 | *Malignant neoplasm of gum |
| 1430 | Malignant neoplasm of upper gum |
| 1431 | Malignant neoplasm of lower gum |
| 1438 | Malignant neoplasm of other sites of gum |
| 1439 | Malignant neoplasm of gum, unspecified |
| 144 | *Malignant neoplasm of floor of mouth |
| 1440 | Malignant neoplasm of floor of mouth, anterior portion |
| 1441 | Malignant neoplasm of floor of mouth, lateral portion |
| 1448 | Malignant neoplasm of other sites of floor of mouth |
| 1449 | Malignant neoplasm of floor of mouth, part unspecified |
| 145 | *Malignant neoplasm of other and unspecified parts of mouth |
| 1450 | Malignant neoplasm of cheek mucosa |
| 1451 | Malignant neoplasm of vestibule of mouth |
| 1452 | Malignant neoplasm of hard palate |
| 1453 | Malignant neoplasm of soft palate |
| 1454 | Malignant neoplasm of uvula |
| 1455 | Malignant neoplasm of palate, unspecified |
| 1456 | Malignant neoplasm of retromolar area |
| 1458 | Malignant neoplasm of other specified parts of mouth |
| 1459 | Malignant neoplasm of mouth, unspecified |
| 146 | *Malignant neoplasm of oropharynx |
| 1460 | Malignant neoplasm of tonsil |
| 1461 | Malignant neoplasm of tonsillar fossa |
| 1462 | Malignant neoplasm of tonsillar pillars (anterior) (posterior) |
| 1463 | Malignant neoplasm of vallecula |
| 1464 | Malignant neoplasm of anterior aspect of epiglottis |
| 1465 | Malignant neoplasm of junctional region of oropharynx |
| 1466 | Malignant neoplasm of lateral wall of oropharynx |
| 1467 | Malignant neoplasm of posterior wall of oropharynx |
| 1468 | Malignant neoplasm of other specified sites of oropharynx |
| 1469 | Malignant neoplasm of oropharynx, unspecified |
| 147 | *Malignant neoplasm of nasopharynx |
| 1470 | Malignant neoplasm of superior wall of nasopharynx |
| 1471 | Malignant neoplasm of posterior wall of nasopharynx |
| 1472 | Malignant neoplasm of lateral wall of nasopharynx |
| 1473 | Malignant neoplasm of anterior wall of nasopharynx |
| 1478 | Malignant neoplasm of other specified sites of nasopharynx |
| 1479 | Malignant neoplasm of nasopharynx, unspecified |
| 148 | *Malignant neoplasm of hypopharynx |
| 1480 | Malignant neoplasm of postcricoid region of hypopharynx |
| 1481 | Malignant neoplasm of pyriform sinus of hypopharynx |
| 1482 | Malignant neoplasm of aryepiglottic fold, hypopharyngeal aspect |
| 1483 | Malignant neoplasm of posterior hypopharyngeal wall |
| 1488 | Malignant neoplasm of other specified sites of hypopharynx |
| 1489 | Malignant neoplasm of hypopharynx, unspecified |
| 149 | *Malignant neoplasm of other and ill-defined sites within the lip, oral cavity and pharynx |
| 1490 | Malignant neoplasm of pharynx, unspecified |
| 1491 | Malignant neoplasm of Waldeyer's ring |
| 1498 | Malignant neoplasm of other sites within the lip, oral cavity, and pharynx |
| 1499 | Malignant neoplasm of ill-defined sites within the lip, oral cavity, and pharynx |
| 150 | *Malignant neoplasm of esophagus |
| 1500 | Malignant neoplasm of cervical esophagus |
| 1501 | Malignant neoplasm of thoracic esophagus |
| 1502 | Malignant neoplasm of abdominal esophagus |
| 1503 | Malignant neoplasm of upper third of esophagus |
| 1504 | Malignant neoplasm of middle third of esophagus |
| 1505 | Malignant neoplasm of lower third of esophagus |
| 1508 | Malignant neoplasm of other specified part of esophagus |
| 1509 | Malignant neoplasm of esophagus, unspecified |
| 151 | *Malignant neoplasm of stomach |
| 1510 | Malignant neoplasm of cardia of stomach |
| 1511 | Malignant neoplasm of pylorus of stomach |
| 1512 | Malignant neoplasm of pyloric antrum of stomach |
| 1513 | Malignant neoplasm of fundus of stomach |
| 1514 | Malignant neoplasm of body of stomach |
| 1515 | Malignant neoplasm of lesser curvature of stomach, unspecified |
| 1516 | Malignant neoplasm of greater curvature of stomach, unspecified |
| 1518 | Malignant neoplasm of other specified sites of stomach |
| 1519 | Malignant neoplasm of stomach, unspecified |
| 152 | *Malignant neoplasm of small intestine, including duodenum |
| 1520 | Malignant neoplasm of duodenum |
| 1521 | Malignant neoplasm of jejunum |
| 1522 | Malignant neoplasm of ileum |
| 1523 | Malignant neoplasm of Meckel's diverticulum |
| 1528 | Malignant neoplasm of other specified sites of small intestine |
| 1529 | Malignant neoplasm of small intestine, unspecified |
| 153 | *Malignant neoplasm of colon |
| 1530 | Malignant neoplasm of hepatic flexure colon |
| 1531 | Malignant neoplasm of transverse colon |
| 1532 | Malignant neoplasm of descending colon |
| 1533 | Malignant neoplasm of sigmoid colon |
| 1534 | Malignant neoplasm of cecum |
| 1535 | Malignant neoplasm of appendix |
| 1536 | Malignant neoplasm of ascending colon |
| 1537 | Malignant neoplasm of splenic flexure |
| 1538 | Malignant neoplasm of other specified sites of large intestine |
| 1539 | Malignant neoplasm of colon, unspecified |
| 154 | *Malignant neoplasm of rectum, rectosigmoid junction, and anus |
| 1540 | Malignant neoplasm of rectosigmoid junction |
| 1541 | Malignant neoplasm of rectum |
| 1542 | Malignant neoplasm of anal canal |
| 1543 | Malignant neoplasm of anus, unspecified |
| 1548 | Malignant neoplasm of rectum, rectosigmoid junction, and anus, other |
| 155 | *Malignant neoplasm of liver and intrahepatic bile ducts |
| 1550 | Malignant neoplasm of liver, primary |
| 1551 | Malignant neoplasm of intrahepatic bile ducts |
| 1552 | Malignant neoplasm of liver, not specified as primary or secondary |
| 156 | *Malignant neoplasm of gallbladder and extrahepatic bile ducts |
| 1560 | Malignant neoplasm of gallbladder |
| 1561 | Malignant neoplasm of extrahepatic bile ducts |
| 1562 | Malignant neoplasm of Ampulla of Vater |
| 1568 | Malignant neoplasm of other specified sites of gallbladder and extrahepatic bile ducts |
| 1569 | Malignant neoplasm of biliary tract, part unspecified |
| 157 | *Malignant neoplasm of pancreas |
| 1570 | Malignant neoplasm of head of pancreas |
| 1571 | Malignant neoplasm of body of pancreas |
| 1572 | Malignant neoplasm of tail of pancreas |
| 1573 | Malignant neoplasm of pancreatic duct |
| 1574 | Malignant neoplasm of islets of langerhans |
| 1578 | Malignant neoplasm of other specified sites of pancreas |
| 1579 | Malignant neoplasm of pancreas, part unspecified |
| 158 | *Malignant neoplasm of retroperitoneum and peritoneum |
| 1580 | Malignant neoplasm of retroperitoneum |
| 1588 | Malignant neoplasm of specified parts of peritoneum |
| 1589 | Malignant neoplasm of peritoneum, unspecified |
| 159 | *Malignant neoplasm of other and ill-defined sites within the digestive organs and peritoneum |
| 1590 | Malignant neoplasm of intestinal tract, part unspecified |
| 1591 | Malignant neoplasm of spleen, not elsewhere classified |
| 1598 | Malignant neoplasm of other sites of digestive system and intra-abdominal organs |
| 1599 | Malignant neoplasm of ill-defined sites within the digestive organs and peritoneum |
| 160 | *Malignant neoplasm of nasal cavities, middle ear and accessory sinuses |
| 1600 | Malignant neoplasm of nasal cavities |
| 1601 | Malignant neoplasm of auditory tube, middle ear and mastoid air cells |
| 1602 | Malignant neoplasm of maxillary sinus |
| 1603 | Malignant neoplasm of ethmoidal sinus |
| 1604 | Malignant neoplasm of frontal sinus |
| 1605 | Malignant neoplasm of sphenoidal sinus |
| 1608 | Malignant neoplasm of nasal cavities, middle ear, and accessory sinuses, other |
| 1609 | Malignant neoplasm of accessory sinus, unspecified |
| 161 | *Malignant neoplasm of larynx |
| 1610 | Malignant neoplasm of glottis |
| 1611 | Malignant neoplasm of supraglottis |
| 1612 | Malignant neoplasm of subglottis |
| 1613 | Malignant neoplasm of laryngeal cartilages |
| 1618 | Malignant neoplasm of other specified sites of larynx |
| 1619 | Malignant neoplasm of larynx, unspecified |
| 162 | *Malignant neoplasm of trachea, bronchus and lung |
| 1620 | Malignant neoplasm of trachea |
| 1622 | Malignant neoplasm of main bronchus |
| 1623 | Malignant neoplasm of upper lobe, bronchus or lung |
| 1624 | Malignant neoplasm of middle lobe, bronchus or lung |
| 1625 | Malignant neoplasm of lower lobe, bronchus or lung |
| 1628 | Malignant neoplasm of other parts of bronchus or lung |
| 1629 | Malignant neoplasm of bronchus and lung, unspecified |
| 163 | *Malignant neoplasm of pleura |
| 1630 | Malignant neoplasm of parietal pleura |
| 1631 | Malignant neoplasm of visceral pleura |
| 1638 | Malignant neoplasm of other specified sites of pleura |
| 1639 | Malignant neoplasm of pleura, unspecified |
| 164 | *Malignant neoplasm of thymus, heart and mediastinum |
| 1640 | Malignant neoplasm of thymus |
| 1641 | Malignant neoplasm of heart |
| 1642 | Malignant neoplasm of anterior mediastinum |
| 1643 | Malignant neoplasm of posterior mediastinum |
| 1648 | Malignant neoplasm of thymus, heart, and mediastinum, other |
| 1649 | Malignant neoplasm of mediastinum, part unspecified |
| 165 | *Malignant neoplasm of other and ill-defined sites within the respiratory system and intrathoracic organs |
| 1650 | Malignant neoplasm of upper respiratory tract, part unspecified |
| 1658 | Malignant neoplasm of other sites respiratory system and intrathoracic organs |
| 1659 | Malignant neoplasm of ill-defined sites within the respiratory system |
| 170 | *Malignant neoplasm of bone and articular cartilage |
| 1700 | Malignant neoplasm of bones of skull and face, except mandible |
| 1701 | Malignant neoplasm of mandible |
| 1702 | Malignant neoplasm of vertebral column, excluding sacrum and coccyx |
| 1703 | Malignant neoplasm of ribs, sternum and clavicle |
| 1704 | Malignant neoplasm of scapula and long bones of upper limb |
| 1705 | Malignant neoplasm of short bones of upper limb |
| 1706 | Malignant neoplasm of pelvic bones, sacrum and coccyx |
| 1707 | Malignant neoplasm of long bones of lower limb |
| 1708 | Malignant neoplasm of short bones of lower limb |
| 1709 | Malignant neoplasm of bone and articular cartilage, site unspecified |
| 171 | *Malignant neoplasm of connective and other soft tissue |
| 1710 | Malignant neoplasm of connective and other soft tissue of head, face and neck |
| 1712 | Malignant neoplasm of connective and other soft tissue of upper limb, including shoulder |
| 1713 | Malignant neoplasm of connective and other soft tissue of lower limb, including hip |
| 1714 | Malignant neoplasm of connective and other soft tissue of thorax |
| 1715 | Malignant neoplasm of connective and other soft tissue of abdomen |
| 1716 | Malignant neoplasm of connective and other soft tissue of pelvis |
| 1717 | Malignant neoplasm of connective and other soft tissue of trunk, unspecified |
| 1718 | Malignant neoplasm of other specified sites of connective and other soft tissue |
| 1719 | Malignant neoplasm of connective and other soft tissue, site unspecified |
| 172 | *Malignant melanoma of skin |
| 1720 | Malignant melanoma of skin of lip |
| 1721 | Malignant melanoma of skin of eyelid, including canthus |
| 1722 | Malignant melanoma of skin of ear and external auditory canal |
| 1723 | Malignant melanoma of skin of other and unspecified parts of face |
| 1724 | Malignant melanoma of skin of scalp and neck |
| 1725 | Malignant melanoma of skin of trunk, except scrotum |
| 1726 | Malignant melanoma of skin of upper limb including shoulder |
| 1727 | Malignant melanoma of skin of lower limb including hip |
| 1728 | Malignant melanoma of other specified sites of skin |
| 1729 | Malignant melanoma of skin, site unspecified |
| 173 | *Other malignant neoplasm of skin |
| 1730 | Malignant neoplasm of skin of lip |
| 1731 | Malignant neoplasm of skin of eyelid, including canthus |
| 1732 | Malignant neoplasm of skin of ear and external auditory canal |
| 1733 | Malignant neoplasm of skin of other and unspecified parts of face |
| 1734 | Malignant neoplasm of scalp and skin of neck |
| 1735 | Malignant neoplasm of skin of trunk, except scrotum |
| 1736 | Malignant neoplasm of skin of upper limb, including shoulder |
| 1737 | Malignant neoplasm of skin of lower limb, including hip |
| 1738 | Malignant neoplasm of other specified sites of skin |
| 1739 | Malignant neoplasm of skin, site unspecified |
| 174 | *Malignant neoplasm of female breast |
| 1740 | Malignant neoplasm of female breast, nipple and areola |
| 1741 | Malignant neoplasm of female breast, central portion |
| 1742 | Malignant neoplasm of female breast, upper-inner quadrant |
| 1743 | Malignant neoplasm of female breast, lower-inner quadrant |
| 1744 | Malignant neoplasm of female breast, upper-outer quadrant |
| 1745 | Malignant neoplasm of female breast, lower-outer quadrant |
| 1746 | Malignant neoplasm of female breast, axillary tail |
| 1748 | Malignant neoplasm of other specified sites of female breast |
| 1749 | Malignant neoplasm of female breast, unspecified |
| 175 | *Malignant neoplasm of male breast |
| 1750 | Malignant neoplasm of male breast, nipple and areola |
| 1759 | Malignant neoplasm of other and unspecified sites of male breast |
| 176 | *Kaposi's sarcoma |
| 1760 | Kaposi's sarcoma of skin |
| 1761 | Kaposi's sarcoma of soft tissue |
| 1762 | Kaposi's sarcoma of palate |
| 1763 | Kaposi's sarcoma of gastrointestinal sites |
| 1764 | Kaposi's sarcoma of lung |
| 1765 | Kaposi's sarcoma of lymph nodes |
| 1768 | Kaposi's sarcoma of other specified sites |
| 1769 | Kaposi's sarcoma of unspecified |
| 179 | Malignant neoplasm of uterus, part unspecified |
| 180 | *Malignant neoplasm of cervix uteri |
| 1800 | Malignant neoplasm of endocervix |
| 1801 | Malignant neoplasm of exocervix |
| 1808 | Malignant neoplasm of other specified sites of cervix |
| 1809 | Malignant neoplasm of cervix uteri, unspecified |
| 181 | Malignant neoplasm of placenta |
| 182 | *Malignant neoplasm of body of uterus |
| 1820 | Malignant neoplasm of corpus uteri, except isthmus |
| 1821 | Malignant neoplasm of isthmus uteri |
| 1828 | Malignant neoplasm of other specified sites of body of uterus |
| 183 | *Malignant neoplasm of ovary and other uterine adnexa |
| 1830 | Malignant neoplasm of ovary |
| 1832 | Malignant neoplasm of fallopian tube |
| 1833 | Malignant neoplasm of broad ligament |
| 1834 | Malignant neoplasm of parametrium |
| 1835 | Malignant neoplasm of round ligament |
| 1838 | Malignant neoplasm of other specified sites of uterine adnexa |
| 1839 | Malignant neoplasm of uterine adnexa, unspecified |
| 184 | *Malignant neoplasm of other and unspecified female genital organs |
| 1840 | Malignant neoplasm of vagina |
| 1841 | Malignant neoplasm of labia majora |
| 1842 | Malignant neoplasm of labia minora |
| 1843 | Malignant neoplasm of clitoris |
| 1844 | Malignant neoplasm of vulva, unspecified |
| 1848 | Malignant neoplasm of other specified sites of female genital organs |
| 1849 | Malignant neoplasm of female genital organ, site unspecified |
| 185 | Malignant neoplasm of prostate |
| 186 | *Malignant neoplasm of testis |
| 1860 | Malignant neoplasm of undescended testis |
| 1869 | Malignant neoplasm of other and unspecified testis |
| 187 | *Malignant neoplasm of penis and other male genital organs |
| 1871 | Malignant neoplasm of prepuce |
| 1872 | Malignant neoplasm of glans penis |
| 1873 | Malignant neoplasm of body of penis |
| 1874 | Malignant neoplasm of penis, part unspecified |
| 1875 | Malignant neoplasm of epididymis |
| 1876 | Malignant neoplasm of spermatic cord |
| 1877 | Malignant neoplasm of scrotum |
| 1878 | Malignant neoplasm of other specified sites of male genital organs |
| 1879 | Malignant neoplasm of male genital organ, site unspecified |
| 188 | *Malignant neoplasm of bladder |
| 1880 | Malignant neoplasm of trigone of urinary bladder |
| 1881 | Malignant neoplasm of dome of urinary bladder |
| 1882 | Malignant neoplasm of lateral wall of urinary bladder |
| 1883 | Malignant neoplasm of anterior wall of urinary bladder |
| 1884 | Malignant neoplasm of posterior wall of urinary bladder |
| 1885 | Malignant neoplasm of bladder neck |
| 1886 | Malignant ureteric orifice |
| 1887 | Malignant neoplasm of urachus |
| 1888 | Malignant neoplasm of other specified sites of bladder |
| 1889 | Malignant neoplasm of bladder, part unspecified |
| 189 | *Malignant neoplasm of kidney and other and unspecified urinary organs |
| 1890 | Malignant neoplasm of kidney, except pelvis |
| 1891 | Malignant neoplasm of renal pelvis |
| 1892 | Malignant neoplasm of ureter |
| 1893 | Malignant neoplasm of urethra |
| 1894 | Malignant neoplasm of paraurethral glands |
| 1898 | Malignant neoplasm of other specified sites of urinary organs |
| 1899 | Malignant neoplasm of urinary organ, site unspecified |
| 190 | *Malignant neoplasm of eye |
| 1900 | Malignant neoplasm of eyeball, except conjunctiva, cornea, retina and choroid |
| 1901 | Malignant neoplasm of orbit |
| 1902 | Malignant neoplasm of lacrimal gland |
| 1903 | Malignant neoplasm of conjunctiva |
| 1904 | Malignant neoplasm of cornea |
| 1905 | Malignant neoplasm of retina |
| 1906 | Malignant neoplasm of choroid |
| 1907 | Malignant neoplasm of lacrimal duct |
| 1908 | Malignant neoplasm of other specified sites of eye |
| 1909 | Malignant neoplasm of eye, part unspecified |
| 191 | *Malignant neoplasm of brain |
| 1910 | Malignant neoplasm of cerebrum, except lobes and ventricles |
| 1911 | Malignant neoplasm of frontal lobe |
| 1912 | Malignant neoplasm of temporal lobe |
| 1913 | Malignant neoplasm of parietal lobe |
| 1914 | Malignant neoplasm of occipital lobe |
| 1915 | Malignant neoplasm of ventricles |
| 1916 | Malignant neoplasm of cerebellum |
| 1917 | Malignant neoplasm of brain stem |
| 1918 | Malignant neoplasm of other parts of brain |
| 1919 | Malignant neoplasm of brain, unspecified |
| 192 | *Malignant neoplasm of other and unspecified parts of nervous system |
| 1920 | Malignant neoplasm of cranial nerves |
| 1921 | Malignant neoplasm of cerebral meninges |
| 1922 | Malignant neoplasm of spinal cord |
| 1923 | Malignant neoplasm of spinal meninges |
| 1928 | Malignant neoplasm of other specified sites of nervous system |
| 1929 | Malignant neoplasm of nervous system, part unspecified |
| 193 | Malignant neoplasm of thyroid gland |
| 194 | *Malignant neoplasm of other endocrine glands and related structures |
| 1940 | Malignant neoplasm of adrenal gland |
| 1941 | Malignant neoplasm of parathyroid gland |
| 1943 | Malignant neoplasm of pituitary gland and craniopharyngeal duct |
| 1944 | Malignant neoplasm of pineal gland |
| 1945 | Malignant neoplasm of carotid body |
| 1946 | Malignant neoplasm of aortic body and other paraganglia |
| 1948 | Malignant neoplasm of other endocrine glands and related structures |
| 1949 | Malignant neoplasm of endocrine gland, site unspecified |
| 195 | *Malignant neoplasm of other and ill-defined sites |
| 1950 | Malignant neoplasm of other and ill-defined sites of head, face and neck |
| 1951 | Malignant neoplasm of other and ill-defined sites of thorax |
| 1952 | Malignant neoplasm of other and ill-defined sites of abdomen |
| 1953 | Malignant neoplasm of other and ill-defined sites of pelvis |
| 1954 | Malignant neoplasm of other and ill-defined sites of upper limb |
| 1955 | Malignant neoplasm of other and ill-defined sites of lower limb |
| 1958 | Malignant neoplasm of other and ill-defined sites of other specified sites |
| 196 | *Secondary and unspecified malignant neoplasm of lymph nodes |
| 1960 | Secondary and unspecified malignant neoplasm of lymph nodes of head, face, and neck |
| 1961 | Secondary and unspecified malignant neoplasm of intrathoracic lymph nodes |
| 1962 | Secondary and unspecified malignant neoplasm of intra-abdominal lymph nodes |
| 1963 | Secondary and unspecified malignant neoplasm of lymph nodes of axilla and upper limb |
| 1965 | Secondary and unspecified malignant neoplasm of lymph nodes of inguinal and lower limb |
| 1966 | Secondary and unspecified malignant neoplasm of intrapelvic lymph nodes |
| 1968 | Secondary and unspecified malignant neoplasm of lymph nodes of multiple sites |
| 1969 | Secondary and unspecified malignant neoplasm of lymph node |
| 197 | *Secondary malignant neoplasm of respiratory and digestive systems |
| 1970 | Secondary malignant neoplasm of lung |
| 1971 | Secondary malignant neoplasm of mediastinum |
| 1972 | Secondary malignant neoplasm of pleura |
| 1973 | Secondary malignant neoplasm of other respiratory organs |
| 1974 | Secondary malignant neoplasm of small intestine, including duodenum |
| 1975 | Secondary malignant neoplasm of large intestine and rectum |
| 1976 | Malignant neoplasm of retroperitoneum and peritoneum |
| 1977 | Secondary malignant neoplasm of liver |
| 1978 | Secondary malignant neoplasm of other digestive organs and spleen |
| 198 | *Secondary malignant neoplasm of other specified sites |
| 1980 | Secondary malignant neoplasm of kidney |
| 1981 | Secondary malignant neoplasm of other urinary organs |
| 1982 | Secondary malignant neoplasm of skin |
| 1983 | Secondary malignant neoplasm of brain and spinal cord |
| 1984 | Secondary malignant neoplasm of other parts of nervous system |
| 1985 | Secondary malignant neoplasm of bone and bone marrow |
| 1986 | Secondary malignant neoplasm of ovary |
| 1987 | Secondary malignant neoplasm of adrenal gland |
| 1988 | *Secondary malignant neoplasm of other specified sites |
| 19881 | Secondary malignant neoplasm of breast |
| 19882 | Secondary malignant neoplasm of genital organs |
| 19889 | Secondary malignant neoplasm of other specified sites |
| 199 | *Malignant neoplasm without specification of site |
| 1990 | Disseminated malignant neoplasm |
| 1991 | Malignant neoplasm of unspecified site (primary) (secondary) |
| 200 | *Lymphosarcoma and reticulosarcoma |
| 2000 | *Reticulosarcoma |
| 20000 | Reticulosarcoma, unspecified site, extranodal solid organ sites |
| 20001 | Reticulosarcoma, lymph nodes of head, face and neck |
| 20002 | Reticulosarcoma, intrathoracic lymph nodes |
| 20003 | Reticulosarcoma, intra-abdominal lymph nodes |
| 20004 | Reticulosarcoma, lymph nodes of axilla and upper limb |
| 20005 | Reticulosarcoma, lymph nodes of inguinal region and lower limb |
| 20006 | Reticulosarcoma, intrapelvic lymph nodes |
| 20007 | Reticulosarcoma, spleen |
| 20008 | Reticulosarcoma, lymph nodes of multiple sites |
| 2001 | *Lymphosarcoma |
| 20010 | Lymphosarcoma, unspecified site, extranodal and solid organ sites |
| 20011 | Lymphosarcoma, lymph nodes of head, face and neck |
| 20012 | Lymphosarcoma, intrathoracic lymph nodes |
| 20013 | Lymphosarcoma, intra-abdominal lymph nodes |
| 20014 | Lymphosarcoma, lymph nodes of axilla and upper limb |
| 20015 | Lymphosarcoma, lymph nodes of inguinal region and lower limb |
| 20016 | Lymphosarcoma, intrapelvic lymph nodes |
| 20017 | Lymphasarcoma, spleen |
| 20018 | Lymphosarcoma, lymph nodes of multiple sites |
| 2002 | *Burkitt's tumor or lymphoma |
| 20020 | Burkitt's tumor or lymphoma, unspecified site, extranodal solid organ sites |
| 20021 | Burkitt's tumor or lymphoma, lymph nodes of head face and neck |
| 20022 | Burkitt's tumor or lymphoma, intrathoracic lymph nodes |
| 20023 | Burkitt's tumor or lymphoma, intra-abdominal lymph nodes |
| 20024 | Burkitt's tumor or lymphoma, lymph nodes of axilla and upper limb |
| 20025 | Burkitt's tumor or lymphoma, lymph nodes of inguinal region and lower limb |
| 20026 | Burkitt's tumor or lymphoma, intrapelvic lymph nodes |
| 20027 | Burkitt's tumor or lymphoma, spleen |
| 20028 | Burkitt's tumor or lymphoma, lymph nodes of multiple sites |
| 2008 | *Other named variants lymphoma |
| 20080 | Other named variants lymphoma, unspecified site, extranodal solid organ sites |
| 20081 | Other named variants lymphoma, lymph nodes of head face and neck |
| 20082 | Other named variants lymphoma, intrathoracic lymph nodes |
| 20083 | Other named variants lymphoma, intra-abdominal lymph nodes |
| 20084 | Other named variants lymphoma, lymph nodes of axilla and upper limb |
| 20085 | Other named variants lymphoma, lymph nodes of inguinal region and lower limb |
| 20086 | Other named variants lymphoma, intrapelvic lymph nodes |
| 20087 | Other named variants lymphoma, spleen |
| 20088 | Other named variants lymphoma, lymph nodes of multiple sites |
| 201 | *Hodgkin's disease |
| 2010 | *Hodgkin's paragranuloma |
| 20100 | Hodgkin's paragranuloma, unspecified site, extranodal solid organ sites |
| 20101 | Hodgkin's paragranuloma, lymph nodes of head, face and neck |
| 20102 | Hodgkin's paragranuloma, intrathoracic lymph nodes |
| 20103 | Hodgkin's paragranuloma, intra-abdominal lymph nodes |
| 20104 | Hodgkin's paragranuloma, lymph nodes of axilla and upper limb |
| 20105 | Hodgkin's paragranloma, lymph nodes of inguinal region and lower limb |
| 20106 | Hodgkin's paragranuloma, intrapelvic lymph nodes |
| 20107 | Hodgkin's paragranuloma, spleen |
| 20108 | Hodgkin's paragranuloma, lymph nodes of multiple sites |
| 2011 | *Hodgkin's granuloma |
| 20110 | Hodgkin's granuloma, unspecified site, extranodal solid organ sites |
| 20111 | Hodgkin's granuloma, lymph nodes of head, face and neck |
| 20112 | Hodgkin's granuloma, intrathoracic lymph nodes |
| 20113 | Hodgkin's granuloma, intra-abdominal lymph nodes |
| 20114 | Hodgkin's granuloma, lymph nodes of axilla and upper limb |
| 20115 | Hodgkin's granuloma, lymph nodes of inguinal region and lower limb |
| 20116 | Hodgkin's granuloma, intrapelvic lymph nodes |
| 20117 | Hodgkin's granuloma, spleen |
| 20118 | Hodgkin's granyloma, lymph nodes of multiple sites |
| 2012 | *Hodgkin's sarcoma |
| 20120 | Hodgkin's sarcoma, unspecified site, extranodal solid organ sites |
| 20121 | Hodgkin's sarcoma, lymph nodes of head, face, and neck |
| 20122 | Hodgkin's sarcoma, intrathoracic lymph nodes |
| 20123 | Hodgkin's sarcoma, intra-abdominal lymph nodes |
| 20124 | Hodgkin's sarcoma, lymph nodes of axilla and upper limb |
| 20125 | Hedgkin's sarcoma, lymph nodes of inguinal region and lower limb |
| 20126 | Hodgkin's sarcoma, intrapelvic lymph nodes |
| 20127 | Hodgkin's sarcoma, spleen |
| 20128 | Hodgkin's sarcoma, lymph nodes of multiple sites |
| 2014 | *Lymphocytic-histiocytic predominance |
| 20140 | Lymphocytic-histiocytic predominance, unspecified site, extranodal solid organ sites |
| 20141 | Lymphocytic-histiocytic predominance, lymph nodes of head, face, and neck |
| 20142 | Lymphocytic-histiocytic predominance, intrathoracic lymph nodes |
| 20143 | Lymphocytic-histiocytic predominance, intra-abdominal lymph nodes |
| 20144 | Lymphocytic-histiocytic predominance, lymph nodes of axilla and upper limb |
| 20145 | Lymphocytic-histiocytic predominance, lymph nodes of inguinal region and lower limb |
| 20146 | Lymphocytic-histiocytic predominance, intrapelvic lymph nodes |
| 20147 | Lymphocytic-histiocytic predominance, spleen |
| 20148 | Lymphocytic-histiocytic predominance, lymph nodes of multiple sites |
| 2015 | *Nodular sclerosis |
| 20150 | Nodular sclerosis, unspecified site, extranodal solid organ sites |
| 20151 | Nodular sclerosis, lymph nodes of head, face, and neck |
| 20152 | Nodular sclerosis, intrathoracic lymph nodes |
| 20153 | Nodular sclerosis, intra-abdominal lymph nodes |
| 20154 | Nodular sclerosis, lymph nodes of axilla and upper limb |
| 20155 | Nodular sclerosis, lymph nodes of inguinal region and lower limb |
| 20156 | Nodular sclerosis, intrapelvic lymph nodes |
| 20157 | Nodular sclerosis, spleen |
| 20158 | Nodular sclerosis, lymph nodes of multiple sites |
| 2016 | *Mixed cellularity |
| 20160 | Mixed cellularity, unspecified site, extranodal solid organ sites |
| 20161 | Mixed cellularity, lymph nodes of head, face and neck |
| 20162 | Mixed cellularity, intrathroacic lymph nodes |
| 20163 | Mixed cellularity, intra-abdominal lymph nodes |
| 20164 | Mixed cellularity, lymph nodes of axilla and upper limb |
| 20165 | Mixed cellularity, lymph nodes of inguinal region and lower limb |
| 20166 | Mixed cellularity, intrapelvic lymph nodes |
| 20167 | Mixed cellularity, spleen |
| 20168 | Mixed cellularity, lymph nodes of multiple sites |
| 2017 | *Lymphocytic depletion |
| 20170 | Lymphocytic depletion, unspecified site, extranodal solid organ sites |
| 20171 | Lymphocytic depletion, lymph nodes of head, face and neck |
| 20172 | Lymphocytic depletion, intrathoracic lymph nodes |
| 20173 | Llymphocitic depletion, intra-abdominal lymph nodes |
| 20174 | Lymphocytic depletion, lymph nodes of axilla and upper limb |
| 20175 | Lymphocytic depletion, lymph nodes of inguinal region and lower limb |
| 20176 | Lymphocytic depletion, intrapelvic lymph nodes |
| 20177 | Lymphocytic depletion, spleen |
| 20178 | Lymphocytic depletion, lymph nodes of multiple sites |
| 2019 | *Hodgkin's disease, unspecified |
| 20190 | Hodgkin's disease, unspecified, unspecified site, extranodal solid organ sites |
| 20191 | Hodgkin's disease, unspecified, lymph nodes of head, face and neck |
| 20192 | Hodgkin’s disease, unspecified, intrathoracic lymph nodes |
| 20193 | Hodgkin’s disease, unspecified, intra-abdominal lymph nodes |
| 20194 | Hodgkin's disease, unspecified, lymph nodes of axilla and upper limb |
| 20195 | Hodgkin's disease, unspecified, lymph nodes of inguinal region and lower limb |
| 20196 | Hodgkin’s disease, unspecified, intrapelvic lymph nodes |
| 20197 | Hodgkin's disease, unspecified, spleen |
| 20198 | Hodgkin's disease, unspecified, lymph nodes of multiple sites |
| 202 | *Other malignant neoplasms of lymphoid and histiocytic tissue |
| 2020 | *Nodular lymphoma |
| 20200 | Nodular lymphoma, unspecified site, extranodal solid organ sites |
| 20201 | Nodular lymphoma, lymph nodes of head, face and neck |
| 20202 | Nodular lymphoma, intrathoracic lymph nodes |
| 20203 | Nodular lymphoma, intra-abdominal lymph nodes |
| 20204 | Nodular lymphoma, lymph nodes of axilla and upper limb |
| 20205 | Nodular lymphoma, lymph nodes of inguinal region and lower limb |
| 20206 | Nodular lymphoma, intrapelvic lymph nodes |
| 20207 | Nodular lymphoma, spleen |
| 20208 | Nodular lymphoma, lymph nodes of multiple sites |
| 2021 | *Mycosis fungoides |
| 20210 | Mycosis fungoides, unspecified site, extranodal solid organ sites |
| 20211 | Mycosis fungoides, lymph nodes of head, face and neck |
| 20212 | Mycosis fungoides, intrathoracic lymph nodes |
| 20213 | Mycosis fungoides, intra-abdominal lymph nodes |
| 20214 | Mycosis fungoides, lymph nodes of axilla and upper limb |
| 20215 | Mycosis fungoides, lymph nodes of inguinal region and lower limb |
| 20216 | Mycosis fungoides, intrapelvic lymph nodes |
| 20217 | Mycosis fungoides, spleen |
| 20218 | Mycosis fungoides, lymph nodes of multiple sites |
| 2022 | *Sezary's disease |
| 20220 | Sezary's disease, unspecified site, extranodal solid organ sites |
| 20221 | Sezary's disease, lymph nodes of head, face, and neck |
| 20222 | Sezary's disease, intrathoracic lymph nodes |
| 20223 | Sezary's disease, intra-abdominal lymph nodes |
| 20224 | Sezary's disease, lymph nodes of axilla and upper limb |
| 20225 | Sezary's disease, lymph nodes of inguinal region and lower limb |
| 20226 | Sezary's disease, intrapelvic lymph nodes |
| 20227 | Sezary's disease, spleen |
| 20228 | Sezary's disease, lymph nodes of multiple sites |
| 2023 | *Malignant histiocytosis |
| 20230 | Malignant histiocytosis, unspecified site, extranodal solid organ sites |
| 20231 | Malignant histiocytosis, lymph nodes of head, face and neck |
| 20232 | Malignant histilcytosis, intrathoracic lymph nodes |
| 20233 | Malignant histiocytosis, intra-abdominal lymph nodes |
| 20234 | Malignant histiocytosis, lymph nodes of axilla and upper limb |
| 20235 | Malignant histiocytosis, lymph nodes of inguinal region and lower limb |
| 20236 | Malignant histiocytosis, intrapelvic lymph nodes |
| 20237 | Malignant histiocytosis, spleen |
| 20238 | Malignant histiocytosis, lymph nodes of multiple sites |
| 2024 | *Leukemic reticuloendotheliosis |
| 20240 | Leukemic reticuloendotheliosis, unspecified site, extranodal solid organ sites |
| 20241 | Leukemic reticuloendotheliosis, lymph nodes of head, face and neck |
| 20242 | Leukemic reticuloendotheliosis, intrathoracic lymph nodes |
| 20243 | Leukemic reticuloendotheliosis, intra-abdominal lymph nodes |
| 20244 | Leukemic reticuloendotheliosis, lymph nodes of axilla and upper limb |
| 20245 | Lleukemic reticuloendotheliosis, lymph nodes of inguinal region and lower limb |
| 20246 | Leukemic reticuloendotheliosis, intrapelvic lymph nodes |
| 20247 | Leukemic reticuloendotheliosis, spleen |
| 20248 | Leukemic reticuloendotheliosis, lymph nodes of multiple sites |
| 2025 | *Letterer-Siwe disease |
| 20250 | Letterer-Siwe disease, unspecified site, extranodal solid organ sites |
| 20251 | Letterer-Siwe disease, lymph nodes of head, face and neck |
| 20252 | Letterer-Siwe disease, intrathoracic lymph nodes |
| 20253 | Letterer-Siwe disease, intra-abdominal lymph nodes |
| 20254 | Letterer-Siwe disease, lymph nodes of axilla and upper limb |
| 20255 | Letterer-Siwe disease, lymph nodes of inguinal region and lower limb |
| 20256 | Letterer-Siwe disease, intrapelvic lymph nodes |
| 20257 | Letterer-Siwe disease, spleen |
| 20258 | Letterer-Siwe disease, lymph nodes of multiple sites |
| 2026 | *Malignant mast cell tumors |
| 20260 | Malignant mast cell tumors, unspecified site, extranodal solid organ sites |
| 20261 | Malignant mast cell tumors, lymph nodes of head, face and neck |
| 20262 | Malignant mast cell tumors, intrathoracic lymph nodes |
| 20263 | Malignant mast cell tumors, intra-abdominal lymph nodes |
| 20264 | Malignant mast cell tumors, lymph nodes of axilla and upper limb |
| 20265 | Malignant mast cell tumors, lymph nodes of inguinal region and lower limb |
| 20266 | Malignant mast cell tumors, intrapelvic lymph nodes |
| 20267 | Malignant mast cell tumors, spleen |
| 20268 | Malignant mast cell tumors, lymph nodes of multiple sites |
| 2028 | *Other lymphomas |
| 20280 | Other lymphomas, unspecified, extranodal solid organ sites |
| 20281 | Other lymphomas, lymph nodes of head, face and neck |
| 20282 | Other lymphomas, intrathoracic lymph nodes |
| 20283 | Other lymphomas, intra-abdominal lymph nodes |
| 20284 | Other lymphomas, lymph nodes of axilla and upper limb |
| 20285 | Other lymphomas, lymph nodes of inguinal region and lower limb |
| 20286 | Other lymphomas, intrapelvic lymph nodes |
| 20287 | Other lymphomas, spleen |
| 20288 | Other lymphomas, lymph nodes of multiple sites |
| 2029 | *Other and unspecified malignant neoplasms of lymphoid and histiocytic tissue |
| 20290 | Other and unspecified malignant neoplasms of lymphoid and histiocytic tissue, unspecified site, extranodal solid organ sites |
| 20291 | Other and unspecified malignant neoplasms of lymphoid and histiocytic tissue, lymph nodes of head, face and neck |
| 20292 | Other and unspecified malignant neoplasms of lymphoid and histiocytic tissue, intrathoracic lymph nodes |
| 20293 | Other and unspecified malignant neoplasms of lymphoid and histiocytic tissue, intra-abdominal lymph nodes |
| 20294 | Other and unspecified malignant neoplasms of lymphoid and histiocytic tissue, lymph nodes of axilla and upper limb |
| 20295 | Other and unspecified malignant neoplasms of lymphoid and histiocytic tissue, lymph nodes of inguinal region and lower limb |
| 20296 | Other and unspecified malignant neoplasms of lymphoid and histiocytic tissue, intrapelvic lymph nodes |
| 20297 | Other and unspecified malignant neoplasms of lymphoid and histiocytic tissue, spleen |
| 20298 | Other and unspecified malignant neoplasms of lymphoid and histiocytic tissue, lymph nodes of multiple sites |
| 203 | *Multiple myeloma and immunoproliferative neoplasms |
| 2030 | *Multiple myeloma |
| 20300 | Multiple myeloma, without mention of remission |
| 20301 | Multiple myeloma, in remission |
| 2031 | *Plasma cell leukemia |
| 20310 | Plasma cell leukemia, without mention of remission |
| 20311 | Plasma cell leukemia, in remission |
| 2038 | *Other immunoproliferative neoplasms |
| 20380 | Other immunoproliferative neoplasms, without mention of remission |
| 20381 | Other immunoproliferative neoplasms, in remission |
| 204 | *Lymphoid leukemia |
| 2040 | *Acute lymphoid leukemia |
| 20400 | Acute lymphoid leukemia, without mention of remission |
| 20401 | Acute lymphoid leukemia, in remission |
| 2041 | *Chronic lymphoid leukemia |
| 20410 | Chronic lymphoid leukemia, without mention of remission |
| 20411 | Chronic lymphoid leukemia, in remission |
| 2042 | *Subacute lymphoid leukemia |
| 20420 | Subacute lymphoid leukemia, without mention of remission |
| 20421 | Subacute lymphoid leukemia, in remission |
| 2048 | *Other lymphoid leukemia |
| 20480 | Other lymphoid leukemia, without mention of remission |
| 20481 | Other lymphoid leukemia, in remission |
| 2049 | *Unspecified lymphoid leukemia |
| 20490 | Unspecified lymphoid leukemia, without mention of remission |
| 20491 | Unspecified lymphoid leukemia, in remission |
| 205 | *Myeloid leukemia |
| 2050 | *Acute myeloid leukemia |
| 20500 | Acute myeloid leukemia, without mention of remission |
| 20501 | Acute myeloid leukemia, in remission |
| 2051 | *Chronic myeloid leukemia |
| 20510 | Chronic myeloid leukemia, without mention of remission |
| 20511 | Chronic myeloid leukemia, in remission |
| 2052 | *Subacute myeloid leukemia |
| 20520 | Subacute myeloid leukemia, without mention of remission |
| 20521 | Subacute myeloid leukemia, in remission |
| 2053 | *Myeloid sarcoma |
| 20530 | Myeloid sarcoma, without mention of remission |
| 20531 | Myeloid sarcoma, in remission |
| 2058 | *Other myeloid leukemia |
| 20580 | Other myeloid leukemia, without mention of remission |
| 20581 | Other myeloid leukemia, in remission |
| 2059 | *Unspecified myeloid leukemia |
| 20590 | Unspecified myeloid leukemia, without mention of remission |
| 20591 | Unspecified myeloid leukemia, in remission |
| 206 | *Monocytic leukemia |
| 2060 | *Acute monocytic leukemia |
| 20600 | Acute monocytic leukemia, without mention of remission |
| 20601 | Acute monocytic leukemia, in remission |
| 2061 | *Chronic monocytic leukemia |
| 20610 | Chronic monocytic leukemia, without mention of remission |
| 20611 | Chronic monocytic leukemia, in remission |
| 2062 | *Subacute monocytic leukemia |
| 20620 | Subacute monocytic leukemia, without mention of remission |
| 20621 | Subacute monocytic leukemia, in remission |
| 2068 | *Other monocytic leukemia |
| 20680 | Other monocytic leukemia, without mention of remission |
| 20681 | Other monocytic leukemia, in remission |
| 2069 | *Unspecified monocytic leukemia |
| 20690 | Unspecified monocytic leukemia, without mention of remission |
| 20691 | Unspecified monocytic leukemia, in remission |
| 207 | *Other specified leukemia |
| 2070 | *Acute erythremia and erythroleukemia |
| 20700 | Acute erythremia and erythroleukemia, without mention of remission |
| 20701 | Acute erythremia and erythroleukemia, in remission |
| 2071 | *Chronic erythremia |
| 20710 | Chronic erythremia, without mention of remission |
| 20711 | Chronic erythremia, in remission |
| 2072 | *Megakaryocytic leukemia |
| 20720 | Megakaryocytic leukemia, without mention of remission |
| 20721 | Megakaryocytic leukemia, in remission |
| 2078 | *Other specified leukemia |
| 20780 | Other specified leukemia, without mention of remission |
| 20781 | Other specified leukemia, in remission |
| 208 | *Leukemia of unspecified cell type |
| 2080 | *Acute leukemia of unspecified cell type |
| 20800 | Acute leukemia of unspecified cell type, without mention of remission |
| 20801 | Acute leukemia of unspecified cell type, in remission |
| 2081 | *Chronic leukemia of unspecified cell type |
| 20810 | Chronic leukemia of unspecified cell type, without mention of remission |
| 20811 | Chronic leukemia of unspecified cell type, in remission |
| 2082 | *Subacute leukemia of unspecified cell type |
| 20820 | Subacute leukemia of unspecified cell type, without mention of remission |
| 20821 | Subacute leukemia of unspecified cell type, in remission |
| 2088 | *Other leukemia of unspecified cell type |
| 20880 | Other leukemia of unspecified cell type, without mention of remission |
| 20881 | Other leukemia of unspecified cell type, in remission |
| 2089 | *Unspecified leukemia |
| 20890 | Unspecified leukemia, without mention of remission |
| 20891 | Unspecified leukemia, in remission |
| 210 | *Benign neoplasm of lip, oral cavity and pharynx |
| 2100 | Benign neoplasm of lip |
| 2101 | Benign neoplasm of tongue |
| 2102 | Benign neoplasm of major salivary glands |
| 2103 | Benign neoplasm of floor of mouth |
| 2104 | Benign neoplasm of other and unspecified parts of mouth |
| 2105 | Benign neoplasm of tonsil |
| 2106 | Benign neoplasm of other parts of oropharynx |
| 2107 | Benign neoplasm of nasopharynx |
| 2108 | Benign neoplasm of hypopharynx |
| 2109 | Benign neoplasm of pharynx, unspecified |
| 211 | *Benign neoplasm of other parts of digestive system |
| 2110 | Benign neoplasm of esophagus |
| 2111 | Benign neoplasm of stomach |
| 2112 | Benign neoplasm of duodenum, jejunum, and ileum |
| 2113 | Benign neoplasm of colon |
| 2114 | Benign neoplasm of rectum and anal canal |
| 2115 | Benign neoplasm of liver and biliary passages |
| 2116 | Benign neoplasm of pancreas, except islets of Langerhans |
| 2117 | Benign neoplasm of islets of Langerhans |
| 2118 | Benign neoplasm of retroperitoneum and peritoneum |
| 2119 | Benign neoplasm of other and unspecified site of digestive system |
| 212 | *Benign neoplasm of respiratory and intrathoracic organs |
| 2120 | Benign neoplasm of nasal cavities, middle ear and accessory sinuses |
| 2121 | Benign neoplasm of larynx |
| 2122 | Benign neoplasm of trachea |
| 2123 | Benign neoplasm of bronchus and lung |
| 2124 | Benign neoplasm of pleura |
| 2125 | Benign neoplasm of mediastinum |
| 2126 | Benign neoplasm of thymus |
| 2127 | Benign neoplasm of heart |
| 2128 | Benign neoplasm of other specified sites of respiratory and intrathoracic organs |
| 2129 | Benign neoplasm of respiratory and intrathoracic organs, site unspecified |
| 213 | *Benign neoplasm of bone and articular cartilage |
| 2130 | Benign neoplasm of bones of skull and face |
| 2131 | Benign neoplasm of lower jaw bone |
| 2132 | Benign neoplasm of vertebral column, excluding sacrum and coccyx |
| 2133 | Benign neoplasm of ribs, sternum and clavicle |
| 2134 | Benign neoplasm of scapula and long bones of upper limb |
| 2135 | Benign neoplasm of short bones of upper limb |
| 2136 | Benign neoplasm of pelvic bones, sacrum and coccyx |
| 2137 | Benign neoplasm of long bones of lower limb |
| 2138 | Benign neoplasm of short bones of lower limb |
| 2139 | Benign neoplasm of bone and articular cartilage, site unspecified |
| 214 | *Lipoma |
| 2140 | Lipoma of skin and subcutaneous tissue of face |
| 2141 | Lipoma of other skin and subcutaneous tissue |
| 2142 | Lipoma of intrathoracic organs |
| 2143 | Lipoma of intra-abdominal organs |
| 2144 | Lipoma of spermatic cord |
| 2148 | Lipoma of other specified sites |
| 2149 | Lipoma, unspecified site |
| 215 | *Other benign neoplasm of connective and other soft tissue |
| 2150 | Benign neoplasm of connective and other soft tissue of head, face and neck |
| 2152 | Benign neoplasm of connective and other soft tissue of upper limb, including shoulder |
| 2153 | Benign neoplasm of connective and other soft tissue of lower limb, including hip |
| 2154 | Benign neoplasm of connective and other soft tissue of thorax |
| 2155 | Benign neoplasm of connective and other soft tissue of abdomen |
| 2156 | Benign neoplasm of connective and other soft tissue of pelvis |
| 2157 | Benign neoplasm of connective and other soft tissue of trunk, unspecified |
| 2158 | Benign neoplasm of connective and other soft tissue of other specified sites |
| 2159 | Benign neoplasm of connective and other soft tissue, site unspecified |
| 216 | *Benign neoplasm of skin |
| 2160 | Benign neoplasm of skin of lip |
| 2161 | Benign neoplasm of eyelid, including canthus |
| 2162 | Benign neoplasm of skin of ear and external auditory canal |
| 2163 | Benign neoplasm of skin of other and unspecified parts of face |
| 2164 | Benign neoplasm of scalp and skin of neck |
| 2165 | Benign neoplasm of skin of trunk, except scrotum |
| 2166 | Benign neoplasm of skin of upper limb, including shoulder |
| 2167 | Benign neoplasm of skin of lower limb, including hip |
| 2168 | Benign neoplasm of other specified sites of skin |
| 2169 | Benign neoplasm of skin, site unspecified |
| 217 | Benign neoplasm of breast |
| 218 | *Uterine leiomyoma |
| 2180 | Submucous leiomyoma of uterus |
| 2181 | Intramural leiomyoma of uterus |
| 2182 | Subserous leiomyoma of uterus |
| 2189 | Leiomyoma of uterus, unspecified |
| 219 | *Other benign neoplasm of uterus |
| 2190 | Benign neoplasm of cervix uteri |
| 2191 | Benign neoplasm of corpus uteri |
| 2198 | Benign neoplasm of other specified parts of uterus |
| 2199 | Benign neoplasm of uterus, part unspecified |
| 220 | Benign neoplasm of ovary |
| 221 | *Benign neoplasm of other female genital organs |
| 2210 | Benign neoplasm of fallopian tube and uterine ligaments |
| 2211 | Benign neoplasm of vagina |
| 2212 | Benign neoplasm of vulva |
| 2218 | Benign neoplasm f other specified sites of female genital organs |
| 2219 | Benign neoplasm of female genital organ, site unspecified |
| 222 | *Benign neoplasm of male genital organs |
| 2220 | Benign neoplasm of testis |
| 2221 | Benign neoplasm of penis |
| 2222 | Benign neoplasm of prostate |
| 2223 | Benign neoplasm of epididymis |
| 2224 | Benign neoplasm of scrotum |
| 2228 | Benign neoplasm of other specified sites of male genital organs |
| 2229 | Benign neoplasm of male genital organs, site unspecified |
| 223 | *Benign neoplasm of kidney and other urinary organs |
| 2230 | Benign neoplasm of kidney, except pelvis |
| 2231 | Benign neoplasm of renal pelvis |
| 2232 | Benign neoplasm of ureter |
| 2233 | Benign neoplasm of bladder |
| 2238 | *Benign neoplasm of other specified sites of urinary organs |
| 22381 | Benign neoplasm of urethra |
| 22389 | Benign neoplasm of other specified sites of urinary organs |
| 2239 | Benign neoplasm of urinary organs, site unspecified |
| 224 | *Benign neoplasm of eye |
| 2240 | Benign neoplasm of eyeball, except conjunctiva, cornea, retina and choroid |
| 2241 | Benign neoplasm of orbit |
| 2242 | Benign neoplasm of lacrimal gland |
| 2243 | Benign neoplasm of conjunctiva |
| 2244 | Benign neoplasm of cornea |
| 2245 | Benign neoplasm of retina |
| 2246 | Benign neoplasm of choroid |
| 2247 | Benign neoplasm of lacrimal duct |
| 2248 | Benign neoplasm of other specified parts of eye |
| 2249 | Benign neoplasm of eye, part unspecified |
| 225 | *Benign neoplasm of brain and other parts of nervous system |
| 2250 | Benign neoplasm of brain |
| 2251 | Benign neoplasm of cranial nerves |
| 2252 | Benign neoplasm of cerebral meninges |
| 2253 | Benign neoplasm of spinal cord |
| 2254 | Benign neoplasm of spinal meninges |
| 2258 | Benign neoplasm of other specified sites of nervous system |
| 2259 | Benign neoplasm of nervous system, part unspecified |
| 226 | Benign neoplasm of thyroid glands |
| 227 | *Benign neoplasm of other endocrine glands and related structures |
| 2270 | Benign neoplasm of suprarenal gland |
| 2271 | Benign neoplasm of parathyroid gland |
| 2273 | Benign neoplasm of pituitary gland and craniopharyngeal duct (pouch) |
| 2274 | Benign neoplasm of pineal gland |
| 2275 | Benign neoplasm of carotid body |
| 2276 | Benign neoplasm of aortic body and other paraganglia |
| 2278 | Benign neoplasm of other endocrine glands and related structures |
| 2279 | Benign neoplasm of endocrine gland, site unspecified |
| 228 | *Hemangioma and lymphangioma, any site |
| 2280 | *Hemangioma, any site |
| 22800 | Hemangioma of unspecified site |
| 22801 | Hemangioma of skin and subcutaneous tissue |
| 22802 | Hemangioma of intracranial structures |
| 22803 | Hemangioma of retina |
| 22804 | Hemangioma of intra-abdominal structures |
| 22809 | Hemangioma, other sites |
| 2281 | Lymphangioma, any site |
| 229 | *Benign neoplasm of other and unspecified sites |
| 2290 | Benign neoplasm of lymph nodes |
| 2298 | Benign neoplasm of other specified sites |
| 2299 | Benign neoplasm, site unspecified |
| 230 | *Carcinoma in situ of digestive organs |
| 2300 | Carcinoma in situ of lip, oral cavity and pharynx |
| 2301 | Carcinoma in situ of esophagus |
| 2302 | Carcinoma in situ of stomach |
| 2303 | Carcinoma in situ of colon |
| 2304 | Carcinoma in situ of rectum |
| 2305 | Carcinoma in situ of anal canal |
| 2306 | Carcinoma in situ of anus, unspecified |
| 2307 | Carcinoma in situ of other and unspecified parts of intestine |
| 2308 | Carcinoma in situ of liver and biliary system |
| 2309 | Carcinoma in situ of other and unspecified digestive organs |
| 231 | *Carcinoma in situ of respiratory system |
| 2310 | Carcinoma in situ of larynx |
| 2311 | Carcinoma in situ of trachea |
| 2312 | Carcinoma in situ of bronchus and lung |
| 2318 | Carcinoma in situ of other specified parts of respiratory system |
| 2319 | Carcinoma in situ of respiratory system, part unspecified |
| 232 | *Carcinoma in situ of skin |
| 2320 | Carcinoma in situ of skin of lip |
| 2321 | Carcinoma in situ of eyelid, including canthus |
| 2322 | Carcinoma in situ of ear and external auditory canal |
| 2323 | Carcinoma in situ of skin of other and unspecified parts of face |
| 2324 | Carcinoma in situ of scalp and skin of neck |
| 2325 | Carcinoma in situ of skin of trunk, except scrotum |
| 2326 | Carcinoma in situ of skin of upper limb, including shoulder |
| 2327 | Carcinoma in situ of skin of lower limb, including hip |
| 2328 | Carcinoma in situ of other specified sites of skin |
| 2329 | Carcinoma in situ of skin, site unspecified |
| 233 | *Carcinoma in situ of breast and genitourinary system |
| 2330 | Carcinoma in situ of breast |
| 2331 | Carcinoma in situ of cervix uteri |
| 2332 | Carcinoma in situ of other and unspecified parts of uterus |
| 2333 | Carcinoma in situ of other and unspecified female genital organs |
| 2334 | Carcinoma in situ of prostate |
| 2335 | Carcinoma in situ of penis |
| 2336 | Carcinoma in situ of other and unspecified male genital organs |
| 2337 | Carcinoma in situ of bladder |
| 2339 | Carcinoma in situ of other and unspecified urinary organs |
| 234 | *Carcinoma in situ of other and unspecified sites |
| 2340 | Carcinoma in situ of eye |
| 2348 | Carcinoma in situ of other specified sites |
| 2349 | Carcinoma in situ, site unspecified |
| 235 | *Neoplasm of uncertain behavior of digestive and respiratory systems |
| 2350 | Neoplasm of uncertain behavior of major salivary glands |
| 2351 | Neoplasm of uncertain behavior of lip, oral cavity and pharynx |
| 2352 | Neoplasm of uncertain behavior of stomach, intestines and rectum |
| 2353 | Neoplasm of uncertain behavior of liver and biliary passage |
| 2354 | Neoplasm of uncertain behavior of retroperitoneum and peritoneum |
| 2355 | Neoplasm of uncertain behavior of other and unspecified digestive organs |
| 2356 | Neoplasm of uncertain behavior of larynx |
| 2357 | Neoplasm of uncertain behavior of trachea, bronchus and lung |
| 2358 | Neoplasm of uncertain behavior of pleura, thymus and mediastinum |
| 2359 | Neoplasm of uncertain behavior of other and unspecified respiratory organs |
| 236 | *Neoplasm of uncertain behavior of genitourinary organs |
| 2360 | Neoplasm of uncertain behavior of uterus |
| 2361 | Neoplasm of uncertain behavior of placenta |
| 2362 | Neoplasm of uncertain behavior of ovary |
| 2363 | Neoplasm of uncertain behavior of other and unspecified female genital organs |
| 2364 | Neoplasm of uncertain behavior of testis |
| 2365 | Neoplasm of uncertain behavior of prostate |
| 2366 | Neoplasm of uncertain behavior of other and unspecified male genital organs |
| 2367 | Neoplasm of uncertain behavior of bladder |
| 2369 | *Neoplasm of uncertain behavior of other and unspecified urinary organs |
| 23690 | Neoplasm of uncertain behavior of urinary organ, unspecified |
| 23691 | Neoplasm of uncertain behavior of kidney and ureter |
| 23699 | Neoplasm of uncertain behavior of other urinary organs |
| 237 | *Neoplasm of uncertain behavior of endocrine glands and nervous system |
| 2370 | Neoplasm of uncertain behavior of pituitary gland and craniopharyngeal duct |
| 2371 | Neoplasm of uncertain behavior of pineal gland |
| 2372 | Neoplasm of uncertain behavior of adrenal gland |
| 2373 | Neoplasm of uncertain behavior of paraganglia |
| 2374 | Neoplasm of uncertain behavior of other and unspecified endocrine glands |
| 2375 | Neoplasm of uncertain behavior of brain and spinal cord |
| 2376 | Neoplasm of uncertain behavior of meninges |
| 2377 | *Neoplasm of uncertain behavior of neurofibromatosis |
| 23770 | Neurofibromatosis, unspecified |
| 23771 | Neurofibromatosis, Type Ⅰ﹝von Recklinghausen's disease﹞ |
| 23772 | Neurofibromatosis, Type Ⅱ[acoustic neurofibromatosis] |
| 2379 | Neoplsm of uncertain behavior of other and unspecified parts of nervous system |
| 238 | *Neoplasm of uncertain behavior of other and unspecified sites and tissues |
| 2380 | Neoplasm of uncertain behavior of bone and articular cartilage |
| 2381 | Neoplasm of uncertain behavior of connective and other soft tissue |
| 2382 | Neoplasm of uncertain behavior of skin |
| 2383 | Neoplasm of uncertain behavior of breast |
| 2384 | Neoplasm of uncertain behavior of polycythemia vera |
| 2385 | Neoplasm of uncertain behavior of histiocytic and mast cells |
| 2386 | Neoplasm of uncertain behavior of plasma cells |
| 2387 | Neoplasm of uncertain behavior of other lymphatic and hematopoietic tissues |
| 2388 | Neoplasm of uncertain behavior of other specified sites |
| 2389 | Neoplasm of uncertain behavior, site unspecified |
| 239 | *Neoplasm of unspecified nature |
| 2390 | Neoplasm of unspecified nature of digestive system |
| 2391 | Neoplasm of unspecified nature of respiratory system |
| 2392 | Neoplasm of unspecified nature of bone, soft tissue and skin |
| 2393 | Neoplasm of unspecified nature of breast |
| 2394 | Neoplasm of unspecified nature of bladder |
| 2395 | Neoplasm of unspecified nature of other genitourinary organs |
| 2396 | Neoplasm of unspecified nature of brain |
| 2397 | Neoplasm of unspecified nature of endocrine glands, and other parts of nervous system |
| 2398 | Neoplasm of unspecified nature of other specified sites |
| 2399 | Neoplasm of unspecified nature, site unspecified |
