# Supplementary material for: Risk of cancer in patients with genital warts: A nationwide, population-based cohort study in Taiwan
Source: PLoS One. 2017 Aug 14;12(8):e0183183. doi: 10.1371/journal.pone.0183183 (PMC5555692; doi:10.1371/journal.pone.0183183)
Supplement: S2 Table — (DOCX) [file pone.0183183.s002.docx]

| General age | Gender | | | | | | | |
| --- | --- | --- | --- | --- | --- | --- | --- | --- |
|  | Male | | | | Female | | | |
|  | nGW(p-y) [A] | GW(p-y) [B] | GW at age [C] | Incidence Rate [(C/(A+B))×100] | nGW(p-y) [A] | GW(p-y) [B] | GW at age [C] | Incidence Rate [(C/(A+B))×100] |
| 0~4 | 698,253.69 | 408.09 | 11 | 0.0016 | 636,752.46 | 399.54 | 12 | 0.0019 |
| 5~9 | 1,116,963.18 | 1,343.71 | 69 | 0.0062 | 1,022,899.89 | 2,658.74 | 40 | 0.0039 |
| 10~14 | 1,361,930.17 | 4,259.54 | 103 | 0.0075 | 1,274,944.41 | 9,096.78 | 118 | 0.0092 |
| 15~19 | 1,419,124.57 | 9,012.91 | 344 | 0.0241 | 1,414,398.50 | 17,673.51 | 1,270 | 0.0887 |
| 20~24 | 1,506,761.42 | 12,494.36 | 1,279 | 0.0842 | 1,582,927.75 | 18,971.53 | 2,784 | 0.1738 |
| 25~29 | 1,579,125.19 | 11,825.34 | 1,591 | 0.1000 | 1,679,799.65 | 14,456.17 | 2,540 | 0.1499 |
| 30~34 | 1,589,785.35 | 9,366.80 | 1,507 | 0.0942 | 1,651,348.98 | 10,235.84 | 1,821 | 0.1096 |
| 35~39 | 1,561,541.98 | 6,687.08 | 1,091 | 0.0696 | 1,574,717.29 | 7,502.86 | 1,322 | 0.0836 |
| 40~44 | 1,557,192.43 | 4,774.83 | 804 | 0.0515 | 1,529,835.07 | 5,607.38 | 1,004 | 0.0654 |
| 45~49 | 1,485,119.21 | 3,494.94 | 569 | 0.0382 | 1,434,578.35 | 4,244.86 | 714 | 0.0496 |
| 50~54 | 1,273,196.68 | 2,610.81 | 401 | 0.0314 | 1,244,796.75 | 2,869.90 | 510 | 0.0409 |
| 55~59 | 959,968.72 | 1,751.11 | 286 | 0.0297 | 972,313.98 | 1,641.88 | 353 | 0.0362 |
| 60~64 | 698,896.89 | 1,385.67 | 192 | 0.0274 | 745,491.97 | 1,096.05 | 176 | 0.0236 |
| 65~69 | 541,029.62 | 1,144.96 | 160 | 0.0295 | 597,555.51 | 784.13 | 114 | 0.0191 |
| 70~74 | 459,960.25 | 998.10 | 139 | 0.0302 | 490,841.86 | 569.48 | 98 | 0.0199 |
| 75~79 | 346,471.30 | 691.36 | 129 | 0.0372 | 348,548.37 | 292.14 | 66 | 0.0189 |
| 80~84 | 206,959.76 | 312.66 | 56 | 0.0270 | 210,201.99 | 133.63 | 21 | 0.0100 |
| 85~89 | 83,665.00 | 122.11 | 34 | 0.0406 | 97,078.50 | 73.48 | 15 | 0.0154 |
| 90~94 | 22,119.10 | 27.36 | 10 | 0.0452 | 31,723.12 | 28.76 | 6 | 0.0189 |
| 95~99 | 3,897.67 | 5.59 | 2 | 0.0512 | 6,734.38 | 2.57 | 2 | 0.0297 |

S2 Table. Age-specific incidence rate of genital warts (GW)

nGW：the number of patient without GW at group of general age

GW：the number of patient with GW at group of general age

GW at age：the number of patient with GW at group of GW age

p-y: person-year
